# Diversification of DNA binding specificities enabled SREBP transcription regulators to expand the repertoire of cellular functions that they govern in fungi

**DOI:** 10.1371/journal.pgen.1007884

**Published:** 2018-12-31

**Authors:** Valentina del Olmo Toledo, Robert Puccinelli, Polly M. Fordyce, J. Christian Pérez

**Affiliations:** 1 Interdisciplinary Center for Clinical Research, University Hospital Würzburg, Würzburg, Germany; 2 Institute for Molecular Infection Biology, University Würzburg, Würzburg, Germany; 3 Department of Genetics, Stanford University, Stanford, California, United States of America; 4 Chan Zuckerberg Biohub, San Francisco, California, United States of America; 5 Department of Bioengineering, Stanford University, Stanford, California, United States of America; 6 Stanford CheM-H Institute, Stanford University, Stanford, California, United States of America; University College Dublin, IRELAND

## Abstract

The Sterol Regulatory Element Binding Proteins (SREBPs) are basic-helix-loop-helix transcription regulators that control the expression of sterol biosynthesis genes in higher eukaryotes and some fungi. Surprisingly, SREBPs do not regulate sterol biosynthesis in the ascomycete yeasts (Saccharomycotina) as this role was handed off to an unrelated transcription regulator in this clade. The SREBPs, nonetheless, expanded in fungi such as the ascomycete yeasts *Candida* spp., raising questions about their role and evolution in these organisms. Here we report that the fungal SREBPs diversified their DNA binding preferences concomitantly with an expansion in function. We establish that several branches of fungal SREBPs preferentially bind non-palindromic DNA sequences, in contrast to the palindromic DNA motifs recognized by most basic-helix-loop-helix proteins (including SREBPs) in higher eukaryotes. Reconstruction and biochemical characterization of the likely ancestor protein suggest that an intrinsic DNA binding promiscuity in the family was resolved by alternative mechanisms in different branches of fungal SREBPs. Furthermore, we show that two SREBPs in the human commensal yeast *Candida albicans* drive a transcriptional cascade that inhibits a morphological switch under anaerobic conditions. Preventing this morphological transition enhances *C*. *albicans* colonization of the mammalian intestine, the fungus’ natural niche. Thus, our results illustrate how diversification in DNA binding preferences enabled the functional expansion of a family of eukaryotic transcription regulators.

## Author summary

Transcription regulation is the primary step by which most cells control the expression of their genes. At its core, this process is mediated by proteins (transcription regulators) that bind to short DNA regulatory elements in a sequence-specific manner. Recent research in multiple model organisms ranging from vertebrates to unicellular yeasts has revealed that evolutionary changes either in the DNA regulatory elements or in the transcription regulators themselves underlie the origin of many traits such as morphological innovations or the ability to colonize new environments. While the effects of mutations that abolish or create DNA regulatory elements are straightforward to rationalize, understanding what sort of modifications the transcription regulators undergo and how these changes impinge upon the regulatory circuitry of the organism remains a key challenge. Here we investigate the mechanisms whereby a family of conserved transcription regulators diversified the biological functions that they control. While in most eukaryotes this family of regulators governs lipid biosynthesis, three members of the family in the human pathogen *Candida albicans* have acquired different functions, some of which contribute to the ability of this yeast to reside in the human host and cause disease.

## Introduction

Evolutionary changes in gene expression patterns constitute a major source of phenotypic diversity [1–4]. The primary step through which all cells regulate expression of their genes is the binding of transcription regulators to *cis*-regulatory sequences. Not surprisingly, gains and losses of *cis*-regulatory sequences have been found to underlie many cases of transcriptional rewiring [5–12]. Although changes in the transcription regulators themselves are also important sources of evolutionary rewiring [13–17], relatively few examples of how these proteins change are understood in molecular detail. In particular, little is known about how different DNA binding preferences arise within a family of transcription regulators and whether such variation results in the functional diversification of the family. We address this question here studying the SREBP (sterol regulatory element binding protein) family of transcription regulators (reviewed in [18–20]). While the SREBPs have been traditionally associated with the regulation of sterol biosynthesis genes, several members of this family appear to govern cellular processes unrelated to lipid synthesis.

SREBPs are basic-helix-loop-helix (bHLH) transcription regulators extensively distributed among eukaryotes. bHLH proteins contain a characteristic 60-to-100-residue DNA binding domain composed of two segments that form amphipathic α-helices separated by a loop region that varies in sequence and length [21, 22]. The SREBPs are unique among the bHLH proteins in that they have a tyrosine residue in a conserved position of the first helix of the DNA binding domain where bHLH proteins normally have an arginine [23, 24]. The tyrosine residue allows the human SREBP to bind, at least *in vitro*, to an additional DNA sequence besides the canonical, palindromic E-box (5’-CANNTG-3’) that is recognized by most bHLH transcription regulators [24]. The significance of this “dual” DNA binding ability remains unclear because chromatin immunoprecipitation (ChIP) experiments have shown that the human SREBP binds *in vivo* to the same canonical, palindromic E-box sequence [25] as other bHLH proteins.

In addition to higher eukaryotes, SREBP family members are also broadly distributed in fungi. While most fungal genomes encode one or two SREBPs, the family has expanded in some lineages such as the *Candida* clade of the ascomycete yeasts (Saccharomycotina). Strikingly, SREBPs do not regulate sterol biosynthesis genes in the ascomycete yeasts as this role was handed off to an unrelated transcription regulator in the common ancestor of all Saccharomycotina [26]. Yet the SREBPs appear to play critical and non-redundant roles in the biology of these fungi. In the human commensal and pathogenic yeast *Candida albicans*, deletion of any of its three SREBPs results in reduced ability to colonize and proliferate in the mammalian host [27–29]. One of the *C*. *albicans* SREBPs (*TYE7*) has been shown to regulate carbohydrate metabolism [27] but the function(s) of the other two regulators is (are) less clear.

Here we investigate the mechanisms that allowed the fungal SREBPs to expand their repertoire of regulatory targets beyond sterol biosynthesis genes. Phylogenetic reconstruction of the family in fungi indicates that the ascomycete yeasts’ SREBPs comprise three distinct branches. We establish that only one of the three branches binds the palindromic DNA E-box motif that SREBPs in higher eukaryotes are known to recognize. The second branch preferentially binds a non-palindromic DNA sequence, whereas the third branch appears to have reduced its DNA binding sequence to a single half-site. Each one of the *C*. *albicans* SREBPs belongs to a different branch of the family, explaining the non-redundant role(s) that each protein has in this organism. Ancestral protein reconstruction experiments indicate that the intrinsic DNA binding plasticity observed in the SREBPs—which is conferred by the characteristic tyrosine residue in the first helix of their DNA binding domain—has been resolved in fungi to give rise to extant proteins that exhibit different DNA binding preferences. Furthermore, we show that in *C*. *albicans* two of its SREBPs act in concert to inhibit a morphological switch under anaerobic conditions. Preventing this morphological transition enhances the fitness of *C*. *albicans* in the mammalian intestine, a natural niche where the fungus resides. Taken together, our results illustrate how generating variation in DNA binding preferences enabled the functional diversification of the SREBP transcription regulators in fungi.

## Results

### Phylogenetic reconstruction of fungal SREBPs

**Fig 1:**
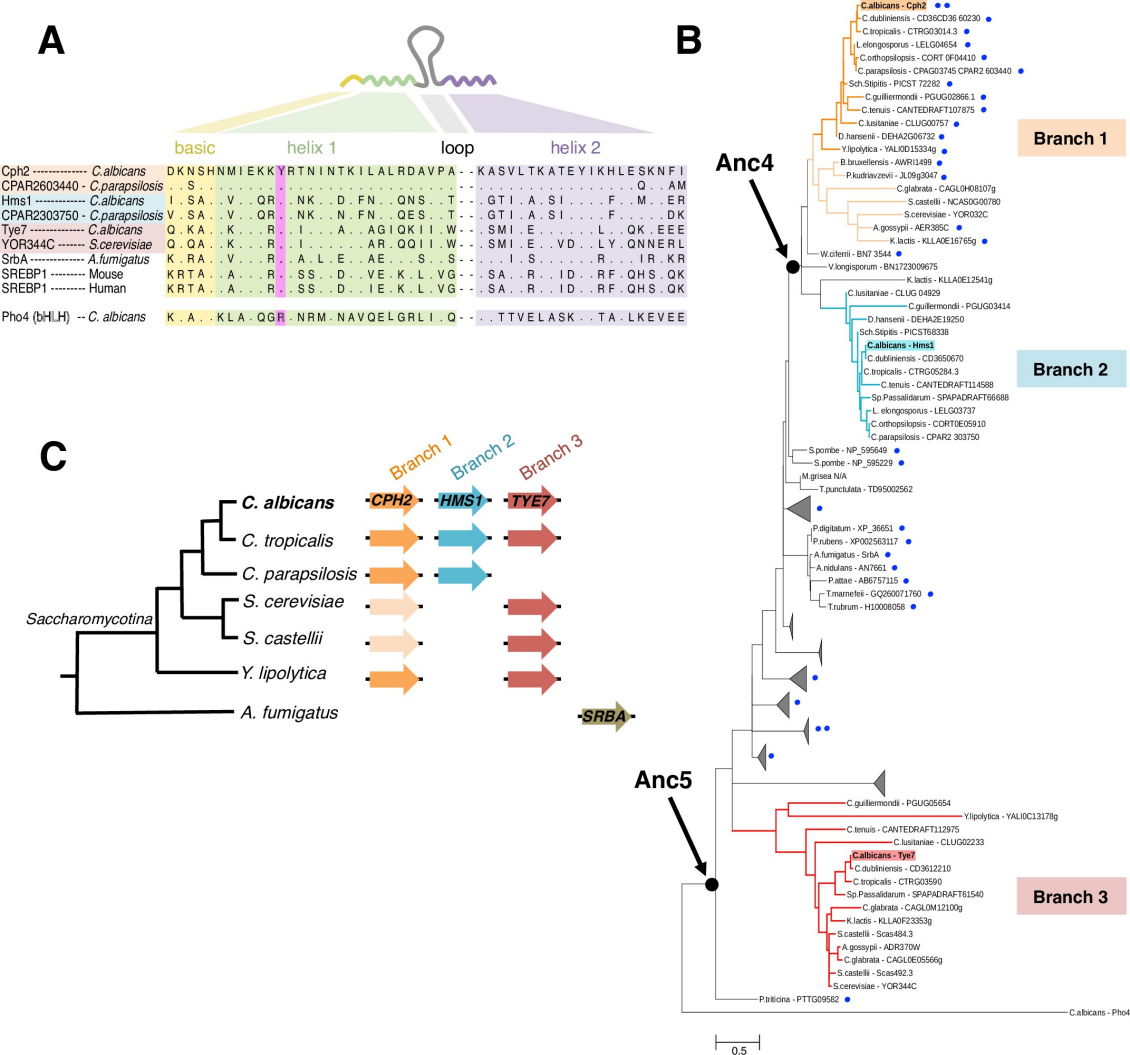
Three branches of the SREBP family of transcription regulators in the ascomycete yeasts (Saccharomycotina). (*A*) Protein alignment of the SREBPs’ DNA binding domain. The SREBPs are basic helix-loop-helix transcription regulators (linear structure drawn on top). Amino acids within the yellow shade belong to the basic region; in green the first helix; and in purple the second helix. The loop region is variable in sequence and length. The tyrosine residue that is the hallmark of the SREBP family is highlighted in pink. Most bHLH regulators (*e*.*g*. Pho4) have a conserved arginine instead of the tyrosine. Dots in the alignment represent the same amino acid residue written at the top of the column. (*B*) Phylogenetic reconstruction of the fungal SREBP family. An alignment of the amino acid sequences of the DNA binding domain of 198 fungal SREBPs was employed to build the phylogeny. Redundant or uninformative sequences are omitted from the tree. Blue dots indicate the presence or absence and number of transmembrane domains in each SREBP. The likely ancestor proteins at the indicated nodes were reconstructed. Highlighted in the tree are three branches. Each branch is represented by a different *C*. *albicans* SREBP: Cph2p (orange), Hms1p (cyan) and Tye7p (red). (*C*) Cladogram depicting the phylogenetic relationships among extant ascomycete yeasts (Saccharomycotina). The genes encoding SREBPs in each species are represented by colored arrows. Same shade of color portrays inferred orthology.

The SREBP family of transcription regulators is widely represented in fungi. A distinctive feature of this family—which distinguishes them from other bHLH proteins—is the presence of a tyrosine residue instead of an arginine in the first helix of the DNA binding domain (Fig 1A). Using this hallmark as the main criterion for inclusion, we assembled a comprehensive phylogeny of the fungal SREBPs based on a manually curated alignment of the DNA binding domain of ~200 proteins (S1 Table; models and computational procedures used for phylogenetic reconstruction are described under Materials and Methods). Little, if any, sequence conservation was detected beyond the SREBPs’ DNA binding domain. Some of the most studied SREBPs (*e*.*g*. those in the model fungus *Schizosaccharomyces pombe* and in humans) harbor transmembrane domains which serve to localize the regulators to intracellular membranes. Upon protein cleavage, the DNA binding domain of these SREBPs is released from the membrane into the cytosol and can shuttle to the nucleus. However, other SREBPs (*e*.*g*. those in the ascomycete model yeast *Saccharomyces cerevisiae*) clearly lack transmembrane domains [30]. Thus, we also scanned the full length of each protein for putative transmembrane sequences to establish whether or not the presence of such domain was widespread across the fungal SREBPs.

The resulting phylogeny points to the existence of several sub-groups within the fungal SREBPs (Fig 1B and S1 Fig). Of particular interest to this report, the ascomycete yeasts’ SREBPs (*i*.*e*. the Saccharomycotina) partitioned in three different branches (labeled 1, 2 and 3 in Fig 1B). The separation in three clusters is also supported by other independent, large-scale reconstructions of fungal gene families such as Fungal Orthogroups [31]. As in most other organisms, the majority of species in the Saccharomycotina encode no more than one or two SREBPs. A few species in the *Candida* clade, however, encode three SREBPs (namely Cph2p, Hms1p and Tye7p) (Fig 1C). Remarkably, each one of these three proteins lies in a different branch of the phylogenetic tree (Fig 1B and 1C) indicating that the *Candida* proteins span a considerable distance in the phylogeny. Within the Saccharomycotina, only branch 1 (which includes the *Candida* Cph2 protein) contains putative transmembrane domains whereas the other two groups (branches 2 and 3 in Fig 1B) do not. A sub-cluster of SREBPs in *Aspergillus* spp. is the only other group outside the Saccharomycotina that appears to lack transmembrane domains. In this study, we focus on characterizing SREBPs that are representative of branches 1, 2 and 3.

### DNA binding preferences across three branches of fungal SREBPs

bHLH proteins are known to recognize and bind to variants of a palindromic DNA sequence termed E-box (the core motif is 5’-CANNTG-3’) [21,25]. Chromatin immunoprecipitation (ChIP) experiments have shown that the archetype member of the SREBP family, the human SREBP1, indeed binds to an instance of this palindromic E-box sequence *in vivo* [25]. However, classic *in vitro* DNA binding assays initially demonstrated that the human SREBP1 can bind not only to the E-box but also to a non-palindromic sequence (5’-TCANNCCA-3’) [23]. Whether binding to this alternative DNA sequence happens only *in vitro* or also takes place *in vivo* remained unclear. Here we sought to evaluate the intrinsic DNA binding preferences of the three selected branches of fungal SREBPs.

**Fig 2:**
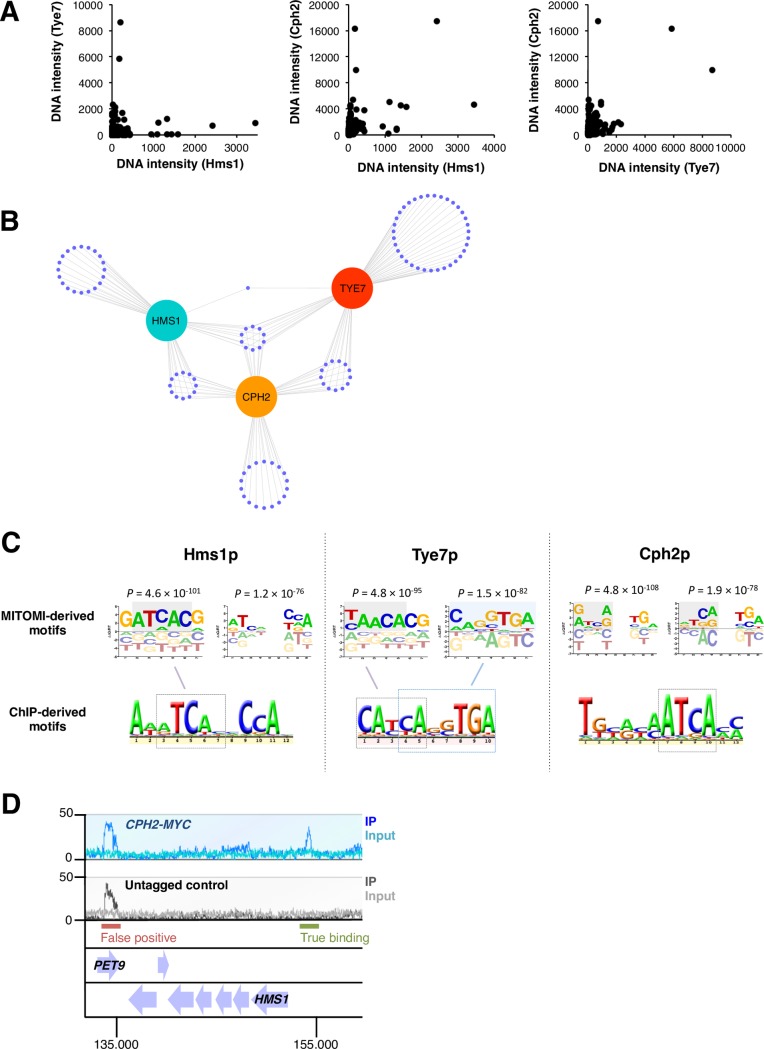
The three branches of ascomycete yeasts’ SREBPs differ from one another in their DNA binding specificities. (*A*) *In vitro* DNA binding preferences of the *C*. *albicans* SREBPs Hms1p, Cph2p and Tye7p in MITOMI, a microfluidics-based approach. Each transcription regulator was evaluated for binding to a set of 740 double-stranded 70-nt oligos designed so that all possible 8-mers were represented. Binding was quantified by measuring the ratio of fluorescence emitted from labeled DNA bound to surface-immobilized labeled regulators. The intensity of binding (DNA intensity) to each oligonucleotide is plotted for each of the three proteins (one dot represents one oligonucleotide). Shown are pairwise comparisons among the regulators. Notice the orthogonal relationship among pairs, particularly between Tye7p and Hms1p. The MITOMI data was derived from two biological replicates. (*B*) Distribution of top 10% of oligonucleotides bound by the *C*. *albicans* SREBPs Hms1p, Cph2p and Tye7p in MITOMI. Each purple dot represents one oligonucleotide. The distances separating the three proteins (cyan, orange and red circles) are inversely proportional to the number of shared oligonucleotides. (*C*) DNA motifs preferred by the *C*. *albicans* SREBPs Hms1p, Cph2p and Tye7p. Shown are representative motifs derived from MITOMI (top) and ChIP (bottom) data sets. Full list of MITOMI motifs with scores (*r*^2^ and P-values) are included in S2 Table. Highlighted is the likely correspondence between MITOMI and ChIP motifs. Notice that the Hms1p MITOMI motif that includes a 2-nucleotide spacer is a very close match to the full Hms1p ChIP motif. The Cph2p MITOMI motifs imply that, *in vitro*, this protein may recognize both ATCANNTGA and ATCANNCCA sequences whereas the ChIP motif suggests binding to the left half-site only (ATCA). The ChIP-derived motif for Cph2p was derived from data included in this report. The ChIP motifs for Tye7 and Hms1p were derived from references [27] and [29], respectively. (*D*) ChIP-Seq analysis of *C*. *albicans* Cph2p. Chromatin immunoprecipitation was carried out with a strain expressing a constitutively active MYC-tagged Cph2p (blue track) and an untagged control strain (grey track). Shown is a 25 kb region of chromosome 5 where a binding event (upstream of the *HMS1* gene) can be visualized and distinguished from a false positive (in the *PET9* gene). 14 binding regions were consistent across replicates and therefore used for the DNA motif analysis displayed in *C*.

As a first systematic approach to establish the DNA binding preferences of the three proteins (Cph2p, Hms1p and Tye7p, all from *Candida albicans*), we employed MITOMI [32], a large-scale microfluidic-based approach that enables the *in vitro* measurement of protein-DNA interactions at equilibrium between transcription regulators and a comprehensive library of oligonucleotides. In each experiment, we assessed binding to a set of 740 double-stranded 70-nt oligos designed so that all possible 8-mers were represented in the library. The binding was then quantified by measuring the ratio of fluorescence emitted from labeled DNA binding to surface-immobilized labeled transcription regulators [32]. Pair-wise comparisons of the oligos bound by the proteins indicate that the oligonucleotide binding patterns observed for Hms1p and Tye7p are largely orthogonal whereas the other two pairs (Cph2p - Hms1p and Cph2p - Tye7p) display some overlapping binding preferences (Fig 2A). Examining the top 10% of oligonucleotides bound by each protein shows that, to a significant extent, they bind different sets of DNA sequences (Fig 2B). A similar pattern emerges if the top 30% or even the top 50% of oligomers are considered (S2 Fig). Comparisons of the top oligonucleotides bound and shared by the proteins reveals that *Ca*Hms1p and *Ca*Tye7p are the most distant from each other whereas *Ca*Cph2p appears as an intermediate (*i*.*e*. it shares a similar number of bound oligomers with *Ca*Hms1p and with *Ca*Tye7p).

We next used MatrixREDUCE [33] to analyze the binding intensities from all oligonucleotides and find DNA motifs overrepresented in the MITOMI data. We run several iterations of the software varying the length of the motif to be searched and allowing or not the inclusion of a 2-nucleotide spacer. All the DNA motifs generated by MatrixREDUCE (at *P* < 1 × 10^−10^) were then compiled and ranked according to *r*^*2*^ and *P*-values (full list with scores can be found in S2 Table). Representatives of the top ranked motifs for each protein are shown in Fig 2C. To a large extent, the MITOMI motifs resembled either the palindromic E-box variant 5’-ATCANNTGA-3’ or the non-palindromic sequence 5’-ATCANNCCA-3’ (or their predicted half-sites). Consistent with the pattern of overlap in bound oligonucleotides, the *Ca*Hms1p- and *Ca*Tye7p-derived motifs were the least similar to each other. *Ca*Cph2p, on the other hand, appeared as an intermediate that could recognize both types of motifs.

As a complementary approach to determine the *in vivo* DNA binding preferences of the proteins, we analyzed genome-wide chromatin immunoprecipitation (ChIP) data. While such datasets have been generated for all three proteins in *C*. *albicans*, a clear DNA motif could be derived only for Tye7p and Hms1p [27, 29]. Thus, we performed our own ChIP-Seq experiment of the third SREBP in *C*. *albicans*, Cph2p. The putative DNA binding domain of Cph2p is located at the N-terminal portion of the protein and is followed by two transmembrane domains that anchor Cph2p to an intracellular membrane. An unidentified signal is thought to trigger the cleavage and release of the N-terminal portion of Cph2p from the membrane and its posterior shuttle to the nucleus [34]. To circumvent the need for an “activating” signal, we generated a *C*. *albicans* strain encoding a truncated version of the protein which ends immediately before the transmembrane domain and is Myc-tagged at this new C-terminus (S3A Fig). The ChIP-Seq experiment conducted with this strain identified 14 high-confidence binding regions located within intergenic sequences (Fig 2D and S3B Fig). A clear DNA motif could be derived from this *in vivo* Cph2p occupancy data set (Fig 2C). The derived motif represents a *bona fide* binding sequence because: First, the purified *Ca*Cph2 protein gel shifted a DNA fragment harboring an instance of the motif; and, second, point mutations introduced in the putative DNA binding site significantly impaired the shift (S3C Fig).

As illustrated in Fig 2C, the MITOMI- and ChIP-derived DNA motifs were, to a significant extent, congruent with each other and revealed distinct DNA binding preferences for each protein. *Ca*Tye7p bound to a singular variant of the palindromic E-box motif that consisted of an extended left half-site (5’-CATCA-3’) and a three-nucleotide right half-site (5’-TGA-3’). While the MITOMI analysis was unable to capture the full 10-nucleotide sequence in a single motif, the two separate Tye7p MITOMI motifs shown in Fig 2C could explain the full-length sequence when combined. *Ca*Hms1p bound to an alternative, non-palindromic sequence (5’-ATCANNCCA-3’). In this case, the Hms1p MITOMI motif that included a 2-nucleotide spacer was in very close agreement with the full Hms1p ChIP motif. In contrast to Hms1p and Tye7p, the Cph2p MITOMI motifs suggested that, at least *in vitro*, this protein may recognize both (5’-ATCANNTGA-3’) and (5’-ATCANNCCA-3’) sequences. The Cph2p ChIP motif, on the other hand, indicated that, *in vivo*, this protein might simply bind to the left portion of either sequence (5’-A/CATCA-3’).

**Fig 3:**
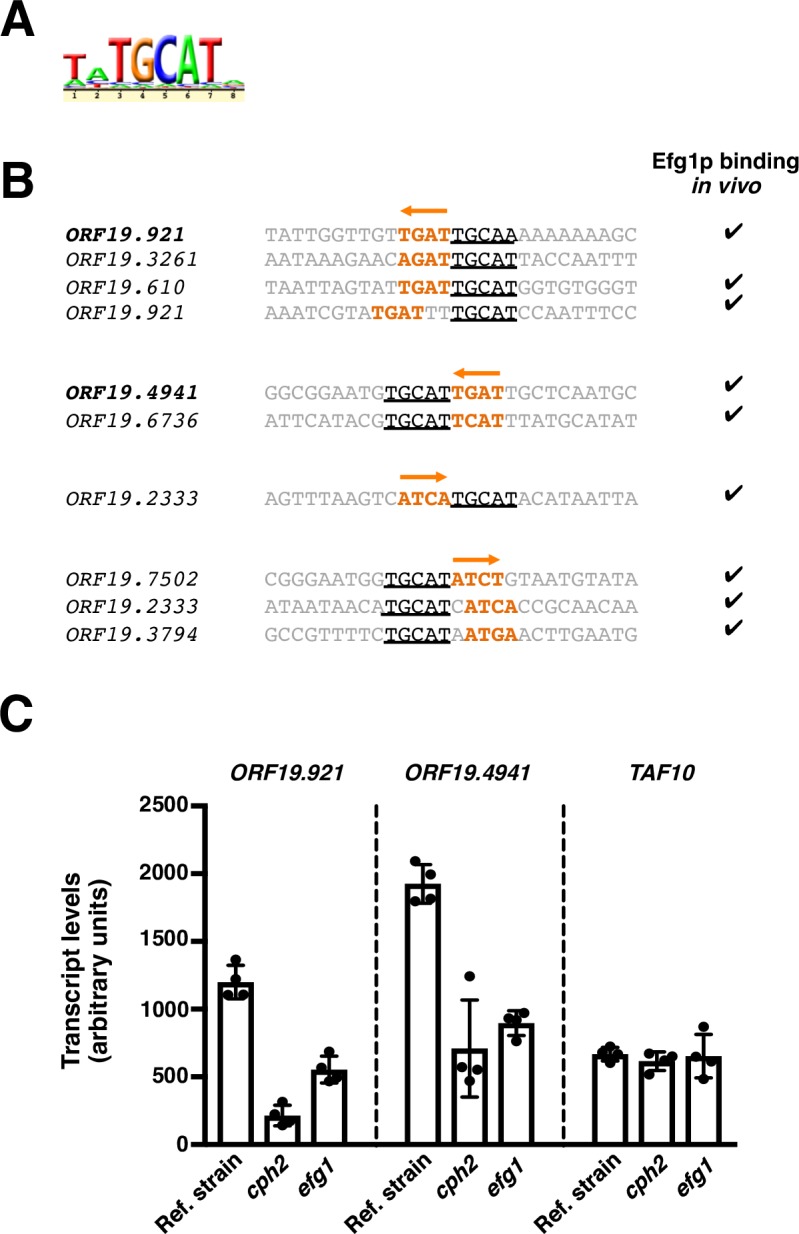
Co-occurrence of Cph2p and Efg1p DNA binding sequences. (*A*) Putative Efg1p motif identified in the set of DNA sequences occupied *in vivo* by Cph2p. (*B*) Distribution of Cph2p and Efg1p DNA binding sites in a subset of the sequences occupied by Cph2p. Putative Cph2p sites are shown in orange whereas the predicted Efg1p sites are underlined. Check marks to the right indicate whether Efg1p has been found to bind *in vivo* to the respective target promoter [35]. (*C*) *CPH2* and *EFG1* co-regulate the expression of target genes. Total RNA was prepared from wild-type, *cph2* and *efg1* deletion mutant strains after a 24-hour incubation under anaerobic conditions (37°C). *ORF19*.*921*, *ORF19*.*4941* and *TAF10* (control) transcript levels were determined by quantitative real-time PCR. Plotted are the mean and SD of four biological replicates. Asterisks denote statistically significant differences compared to the reference strain.

Since manual, detailed examination of the DNA regions occupied by the Cph2 protein *in vivo* produced no evidence of a composite motif (*i*.*e*. a second half-site), we considered the possibility that co-factors could contribute to this protein’s binding *in vivo*. Indeed, DNA motif searches in our *Ca*Cph2 ChIP dataset revealed the co-occurrence of a DNA sequence that closely resembles the DNA motif recognized by the *C*. *albicans* regulator Efg1p (Fig 3A). Consistent with this result, we found that a significant proportion of these sites are occupied by Efg1p *in vivo* (*P* = 2.6 × 10^−5^) (Fig 3B; [35]). These observations suggested that the Cph2 and Efg1 proteins may interact *in vivo* (either by binding cooperatively or by competing with each other for binding) to regulate a subset of target promoters. Consistent with this hypothesis, we found that the expression of two direct targets of regulation (*ORF19*.*921* and *ORF19*.*4941*; each containing Cph2p- and Efg1p-binding sites in their putative promoter regions as indicated in Fig 3B) is dependent, at least in part, on *CPH2* and *EFG1* (Fig 3C). Taken together, these results indicate that *Ca*Cph2p may *by itself* recognize a shorter DNA sequence (compared to the other two branches of SREBPs studied here) but it likely operates in concert with co-factors such as Efg1p.

### Several fungal SREBPs bind preferentially to the non-palindromic 5’-TCANNCCA-3’ motif

**Fig 4:**
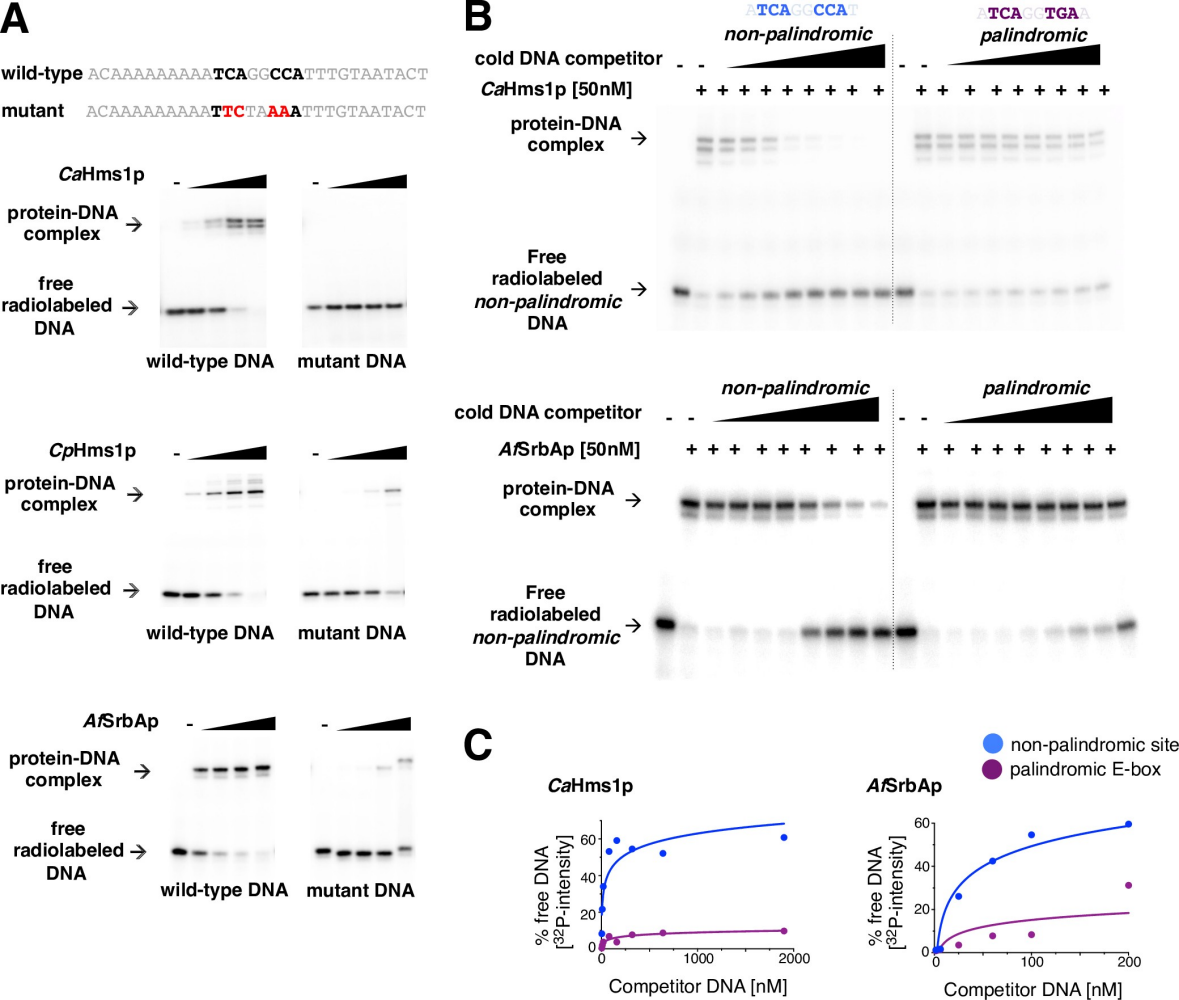
Several fungal SREBPs exhibit an intrinsic DNA binding preference for a non-palindromic DNA motif. (*A*) Gel shift assays showing binding of three fungal SREBPs to a non-palindromic DNA sequence. P^32^-labeled DNA fragments containing the wild-type or mutant non-palindromic binding site were incubated with increasing concentrations (0, 1.56, 6.25, 25 and 100 nM) of the purified DNA binding domain of *C*. *albicans* Hms1p (top), *C*. *parapsilosis* Hms1p (middle) or *A*. *fumigatus* SrbAp (bottom) for 90 min at room temperature in standard EMSA buffer and resolved in 6% polyacrylamide gels run with 0.5× TGE. Point mutations introduced in the DNA binding site are shown in red. (*B*) Competition experiments to determine the DNA binding preferences of the SREBPs *Ca*Hms1p (top) and *Af*SrbAp (bottom). The purified DNA binding domain of either protein was incubated with a P^32^-labeled DNA fragment containing the non-palindromic binding site. Upon binding, increasing concentrations of unlabeled competitor DNA fragments harboring either palindromic E-box or non-palindromic binding sites were added to the reactions, and the mixtures were then resolved by polyacrylamide gel electrophoresis. (*C*) Quantification of competition assays shown in *B*. Plotted is the amount of radiolabeled DNA that becomes protein-free upon addition of unlabeled DNA competitor. Notice that the rapid increase in signal in the blue curve indicates that both proteins show strong preference (at least one order of magnitude) for the non-palindromic DNA sequence.

MITOMI and ChIP data clearly indicate that the Hms1 protein from *C*. *albicans* binds a non-palindromic DNA sequence, which is unusual because most bHLH proteins bind a palindromic DNA motif. We wondered whether this unusual binding preference was exclusive to this protein in this species or extended to other SREBPs. To address this question, in addition to *Ca*Hms1p, we purified the putative DNA binding domains of the *C*. *parapsilosis* Hms1 (CPAR2_303750) and the *A*. *fumigatus* SrbA (Afu2g01260) proteins and carried out electrophoretic mobility gel shift assays (EMSAs). As shown in Fig 4A, all three proteins bound to a DNA fragment harboring an instance of the non-palindromic motif. This binding was specific to the analyzed DNA sequence because point mutations introduced in the putative binding site abolished or severely impaired binding (Fig 4A).

We next wanted to determine whether the proteins were able to discriminate between non-palindromic and canonical E-box sequences. For this, we carried out competition binding assays in which we incubated *Ca*Hms1p or *Af*SrbA with a ^32^P-labeled DNA fragment carrying the non-palindromic sequence. Upon binding, we competed the reactions with unlabeled DNA fragments harboring either the non-palindromic site or the canonical E-box sequence (Fig 4B). The former DNA fragment was a stronger competitor compared to the latter (Fig 4B and 4C and S4 Fig) indicating that the proteins exhibit a marked preference for the non-palindromic sequence. Taken together, these results demonstrate that the preferential binding to a non-palindromic DNA sequence is a property shared by multiple fungal SREBPs, both within and outside the ascomycete yeasts.

### DNA binding specificity in the *C*. *albicans* SREBP Hms1 is conferred by residues in the first helix and loop region of the DNA binding domain

We showed above that the purified DNA binding domain of the *Ca*Hms1 protein exhibits a strong preference for its cognate DNA binding sequence (a non-palindromic DNA site) over the canonical E-box motif in competitive EMSAs (Fig 4B and 4C). Since *Ca*Hms1p’s ability to discriminate between DNA sequences is clearly an intrinsic property of the protein, we sought to determine what portion(s) of its DNA binding domain confer(s) this ability.

**Fig 5:**
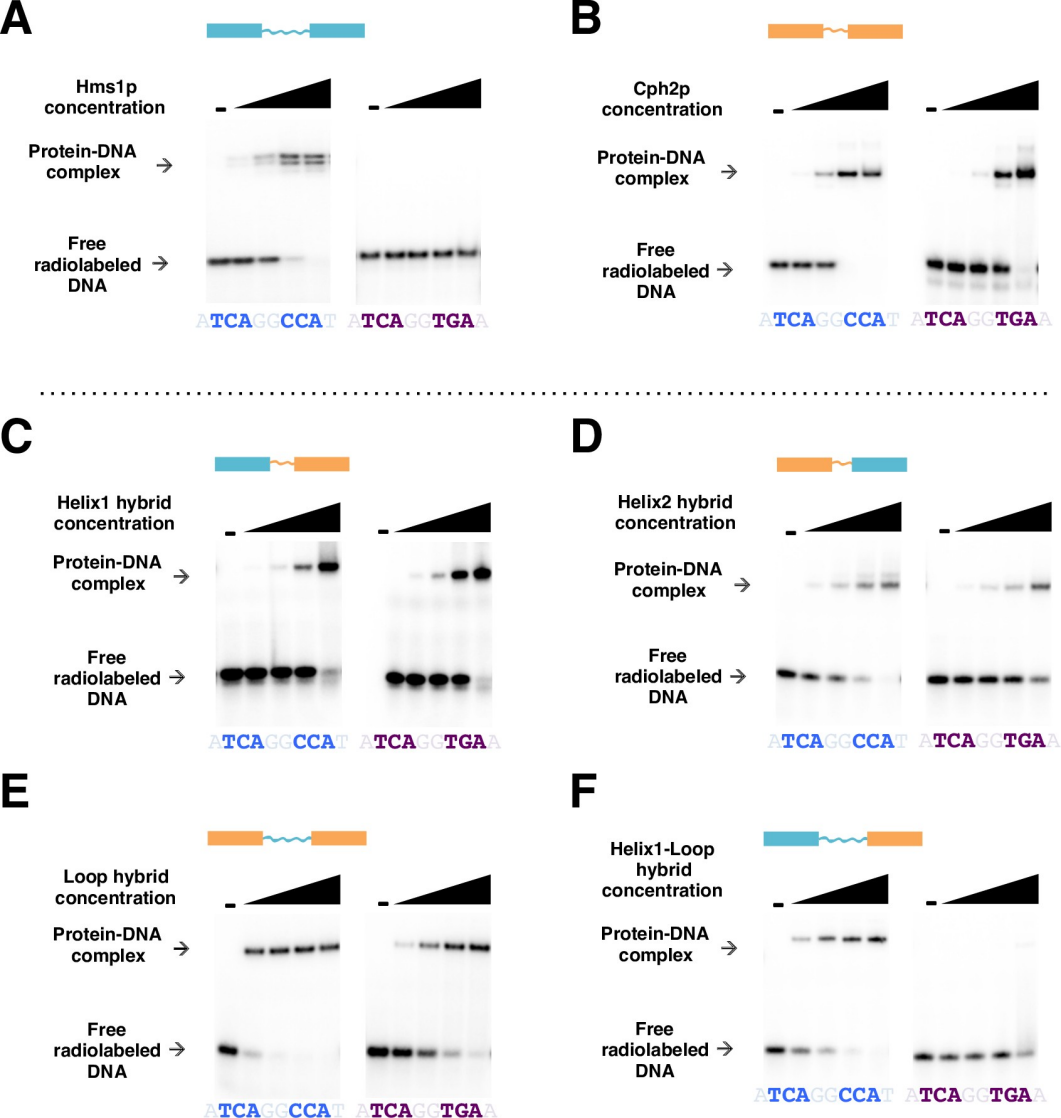
DNA binding specificity in the *C*. *albicans* SREBP Hms1 is conferred by residues in the first helix and loop region of its DNA binding domain. *(A-F)* Gel shift assays to determine the protein regions of the *C*. *albicans* SREBP Hms1p (cyan) that are necessary to discriminate between binding to its cognate DNA binding site (sequence in blue) and binding to the canonical E-box sequence (purple) typically recognized by other SREBPs. Chimeric proteins were generated by exchanging parts of the DNA binding domain of *Ca*Hms1p (cyan) with that of *Ca*Cph2p (orange). The latter protein shows much reduced ability to discriminate *in vitro* between both DNA binding sites (*B*). Chimeric proteins harboring the *Ca*Hms1p first helix (*C*), second helix (*D*) or loop (*E*) alone displayed no discrimination between the two sequences. The chimeric protein containing the *Ca*Hms1p first helix and loop bound preferentially to the Hms1p’s cognate DNA binding sequence (*F*). The protein concentrations evaluated in the gel shifts were 0, 1.56, 6.25, 25 and 100 nM.

We constructed several chimeric proteins by exchanging one of three portions (first helix, loop region or second helix) of the DNA binding domains of *Ca*Hms1p and *Ca*Cph2p (the latter protein displayed little, if any, ability to discriminate *in vitro* between the two DNA sequences evaluated here (S5A and S5B Fig)). We then employed EMSAs to probe each chimeric protein for their ability to bind DNA fragments harboring either the cognate *HMS1* binding site or the canonical E-box sequence. We found that a chimeric protein consisting of the first helix and the loop from *Ca*Hms1p and the second helix from *Ca*Cph2p recapitulated almost completely the ability to discriminate between the two DNA sequences as the native *Ca*Hms1p (Fig 5). Chimeric proteins containing only the first helix or only the loop from *Ca*Hms1p showed little if any discrimination. Therefore, from these experiments we conclude that residues within the first helix combined with residues in the loop region of *Ca*Hms1p confer the ability to bind specifically to the non-palindromic sequence.

### Ancestral protein reconstruction reveals pattern of divergence of SREBP’s DNA binding preferences

A major difference between branches 2 and 3 of the fungal SREBPs (these branches are represented by *Ca*Hms1p and *Ca*Tye7p, respectively) is their intrinsic ability to discriminate between the canonical, palindromic E-box (core motif 5’-CANNTG-3’) and the non-palindromic DNA sequence 5’-ATCANNCCA-3’. While Hms1p and related proteins exhibited a strong preference for the latter (Fig 4B and 4C), Tye7p showed a preference, albeit slight, for the former (S5C and S5D Fig). The presence of a tyrosine residue in the DNA binding domain of the SREBPs (instead of a conserved arginine in other bHLH proteins) allows the promiscuous binding to either DNA sequence [24]. But, how did the preference to bind one or the other sequence come about? At least two scenarios could be envisioned. First, an ancestor that had a clear preference for one of the two sequences could have given rise to a lineage that reduced (and eventually flipped) its DNA binding preference. Alternatively, an ancestor that bound both sequences equally well could have given rise to one branch that tilted its DNA binding preference in one direction and another branch whose DNA binding preference tilted in the opposite direction. To empirically test these models, we used ancestral protein reconstruction [14, 36, 37].

**Fig 6:**
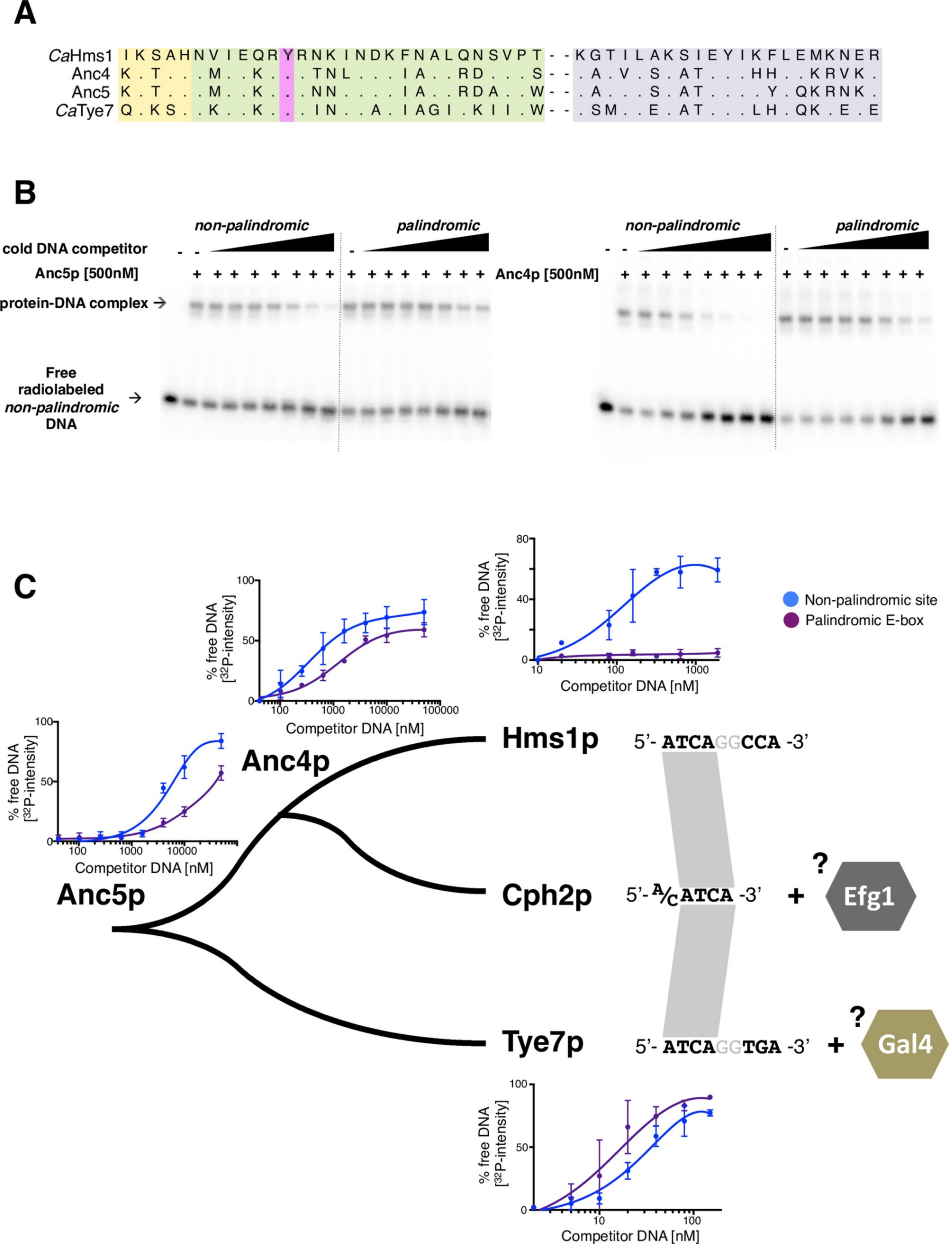
Ancestral protein reconstruction and divergence of DNA binding preferences in fungal SREBPs. (*A*) Protein sequence of the DNA binding domains of the two ancestors that were reconstructed and synthesized (Anc4 and Anc5). The corresponding sequences for the *C*. *albicans* Hms1 and Tye7 proteins are included for comparison. Colour shades represent the same regions of the proteins as in Fig 1A. (*B*) Competition experiments to determine the DNA binding preferences of Anc5p (left panel) and Anc4p (right panel). Assays were carried out as described for Fig 4B. (*C*) Diagram depicting the DNA binding preferences in extant and ancestral fungal SREBPs. Shown are the quantifications of *in vitro* DNA binding competition assays carried out with the two ancestor proteins (gels in Fig 6B), *Ca*Hms1p (gels in Fig 4B) and *Ca*Tye7p (gels in S5C Fig). At least three independent experiments were quantified for each protein and the means ± S.D. are plotted. The more separation between the blue and the purple line indicates clearer preference for one of the two sequences. Notice that Hms1p shows strong preference for the non-palindromic sequence (blue) whereas Tye7 displays slight preference for the palindromic E-box (purple). To the right are shown the DNA sequences that each protein binds *in vivo*. Co-factors such as Efg1p and Gal4p may contribute, at least in part, to the specific binding *in vivo* of Cph2p and Tye7p (discussed in the text).

We inferred the amino acid sequences of the putative ancestors at two selected nodes of the fungal SREBP phylogeny (Figs 1B and 6A and S6 Fig), then expressed and purified these ancestral proteins. We first assayed the ability of these proteins to recognize the canonical E-box and non-palindromic Hms1p binding sequence in gel shift assays. While a higher amount of ancestor protein was required to bind to the DNA sequences and produce a “shift,” the protein-DNA interactions were still sequence-specific (S6C Fig). We then carried out *in vitro* competition assays to determine the preference of the purified ancestral proteins for either the palindromic or the non-palindromic sequence. As shown in Fig 6B and 6C, the “oldest” ancestor (Anc5) displayed a slight preference for the non-palindromic sequence. Similarly, the more “recent” ancestor (Anc4) also showed preference for the same sequence (non-palindromic site over the canonical E-box). However, the preference exhibited by *Ca*Hms1p towards the non-palindromic sequence is still about an order of magnitude higher than what is exhibited by both ancestors (Fig 6C).

These findings support the notion that two extant branches of fungal SREBPs, which are represented by Hms1p and Tye7p, followed divergent paths after separating from their last common ancestor: The Hms1 lineage enhanced the ancestor’s initial preference for the non-palindromic sequence whereas the Tye7 lineage reduced, and eventually flipped, the DNA-binding preference of the ancestor.

### The *C*. *albicans* SREBPs Hms1 and Cph2 constitute a regulatory cascade that prevents yeast-to-filament transition under anaerobic conditions

The fact that the *C*. *albicans* genome harbors three SREBPs—whereas most other organisms have only one or two—raises the question of what functions they perform in this particular fungus. The most prevalent function associated with SREBPs in fungi is the regulation of ergosterol biosynthesis; however, this role was taken over by the unrelated protein Upc2p in the lineage leading to *C*. *albicans* [26]. Each one of the *C*. *albicans* SREBPs seems to play a critical role in the biology of the fungus in the mammalian host because strains deleted for any single SREBP gene have reduced fitness in murine models of *Candida* colonization [27–29]. While *Ca*Tye7p has been implicated in glycolysis and sugar metabolism [27], the function(s) of *Ca*Hms1p and *Ca*Cph2p remain(s) less clear.

**Fig 7:**
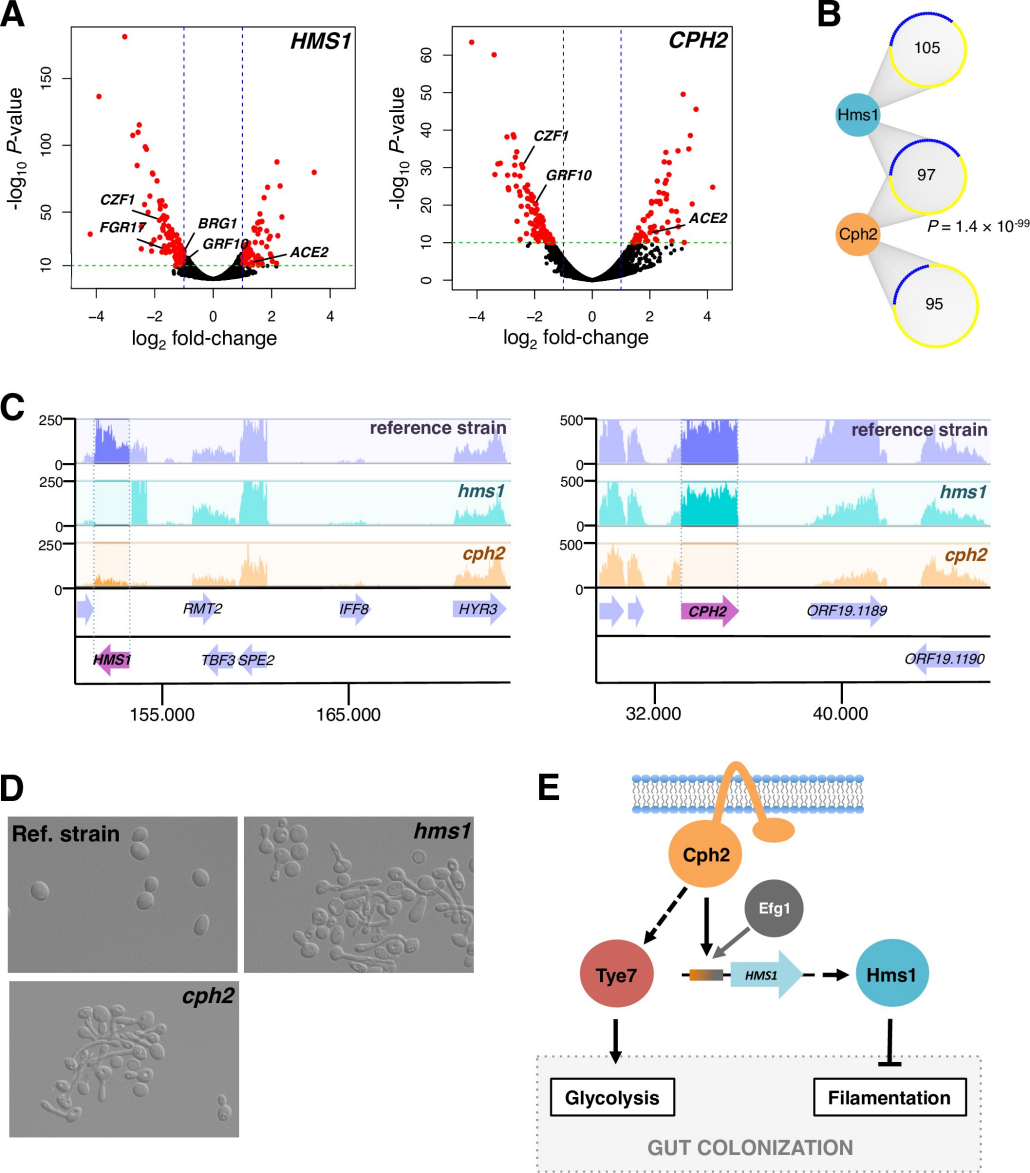
*C*. *albicans* Hms1p and Cph2p form a regulatory cascade that controls a morphological switch under anaerobic conditions. (*A*) Identification of transcripts regulated by *HMS1* and *CPH2*. Total RNA was prepared from wild-type, *hms1* and *cph2* deletion mutant strains grown at 37°C under anaerobic conditions. Shown are volcano plots where each dot represents one transcript. In red are significantly up- or down-regulated transcripts. The regulators of filamentation *ACE2*, *BRG1*, *CZF1*, *FGR17* and *GRF10* are marked. (*B*) Overlap of targets of regulation between Hms1p and Cph2p. Up-regulated genes are shown in yellow and down-regulated genes in blue. The hypergeometric distribution was employed to calculate the significance of the overlap. (*C*) Representative segments of RNA-Seq track for the wild-type reference strain (purple), *hms1* (blue track) and *cph2* (orange track) mutants. Notice that the levels of the *HMS1* transcript are dramatically reduced in the *cph2* deletion strain (left panel). (*D*) *HMS1* and *CPH2* prevent *C*. *albicans* filamentation under anaerobic conditions. The wild-type reference strain, *cph2* and *hms1* mutants were grown in Todd-Hewitt broth at 37°C under anaerobiosis for 24 h. Morphology of cells was examined by microscopy. (*E*) Model depicting the relationships among the three *C*. *albicans* SREBPs.

The SREBPs in other species and the *C*. *albicans* SREBP Tye7 regulate cellular processes sensitive to oxygen [27, 38, 39]. We reasoned, then, that the other two *C*. *albicans* SREBPs might play a role when the fungus proliferates in a niche largely devoid of oxygen. To identify the repertoire of target genes regulated by *HMS1* and *CPH2*, we performed transcriptome analyses (RNA sequencing) of the wild-type reference strain and isogenic *cph2* or *hms1* deletion mutants grown in an anaerobic chamber at 37°C (the temperature of the mammalian host) (Fig 7).

Overall, the RNA-Seq experiment revealed 202 and 192 protein-coding transcripts whose expression was dependent on *CPH2* and *HMS1*, respectively (-log_10_ *P* > 10 and expression changes >2-fold; Fig 7A, S3 Table; 685 and 235 targets at *P* < 0.001 and expression changes >2-fold). There was a significant overlap in targets of regulation between *CPH2* and *HMS1* (*P* = 1.36 × 10^−99^) (Fig 7B and S7 Fig) implying that these two SREBPs form a regulatory cascade. Consistent with this idea, we found that *HMS1* expression is dependent on *CPH2* (but not vice versa) (Fig 7C). Cph2p binds *in vivo* to the intergenic region upstream of *HMS1* (Fig 2D and [34]) further supporting a direct regulatory link between these two factors. Gene Ontology analysis of the differentially expressed genes revealed filamentous growth (*P* = 1.7 × 10^−4^), pathogenesis (*P* = 1.44 × 10^−7^) and biofilm formation (*P* = 1.82 × 10^−13^) as cellular processes or functions enriched in the dataset (it should be noted that over 50% of the genes in the dataset are annotated as having unknown functions). Indeed, the transcript levels of several well-established regulators of yeast-to-filament transition, *e*.*g*. *CZF1*, *GRF10* and *ACE2* [40–42], appeared to be under control of both *HMS1* and *CPH2*. The direction of the change in expression in *CZF1*, *GRF10* and *ACE2* suggested that, under the environmental condition evaluated, both *HMS1* and *CPH2* would work by preventing filamentation. In other growth conditions, *HMS1* and *CPH2* have been associated with the opposite phenotype (*i*.*e*. promoting the yeast-to-filament transition) [43, 44].

To establish whether indeed *HMS1* and *CPH2* work as predicted by our RNA-Seq experiment, we examined the morphology of both deletion mutant strains under anaerobic conditions at 37°C. We found that the *hms1* deletion mutant as well as the *cph2* mutant strain formed filaments while the wild-type reference strain did not (Fig 7D). We have recently demonstrated that filamentation in *C*. *albicans* is detrimental for intestinal colonization [45]. Since the *C*. *albicans hms1* or *cph2* deletion mutant strains are impaired in their ability to persist in the murine gut [28, 29], our results suggest that *HMS1* and *CPH2* may promote gut colonization, at least in part, by preventing the yeast-to-filament morphology transition (Fig 7E).

## Discussion

In this study, we have explored the mechanisms driving the diversification of a eukaryotic transcription regulator family, the SREBPs. In the ascomycete yeasts, the genomes of several *Candida* species encode three SREBPs. Previous work has shown that in this group of fungi, transcription of ergosterol biosynthesis genes—the main function associated with the family in most organisms—is regulated by proteins unrelated to the SREBPs. These observations implied that the family diversified their function in the ascomycete yeasts, *i*.*e*. that the proteins adopted other genes and cellular processes as main targets of regulation. We report that concomitant with a diversification of the cellular functions governed by the SREBPs, these proteins underwent significant changes in their DNA binding specificities. Several lines of evidence support this statement. First, phylogenetic reconstruction of the SREBP family based on the DNA binding domain of the proteins revealed that each one of the three *Candida* SREBPs belongs to a different branch of the family tree (Fig 1), a pattern consistent with these three proteins being non-redundant. Second, the three *Candida* SREBPs displayed, to a significant extent, non-overlapping patterns of binding to a comprehensive library of DNA sequences (Fig 2). And, third, only one of the three SREBPs in *Candida* bound to the palindromic E-box motif which is recognized by most bHLH proteins (Fig 2); in contrast, the *Candida* Hms1p branch exhibited a strong preference for a non-palindromic DNA sequence whereas the third *Candida* SREBP, Cph2p, bound to a sequence consisting of only a half-site motif but likely in conjunction with a co-factor (Figs 2 and 3). The SREBPs played a key role in the regulation of a morphological switch (Fig 7) or in sugar metabolism in *C*. *albicans* [27]; therefore, the diversification in DNA binding specificities appears to be central to the SREBPs’ expansion in targets of regulation in the lineage leading to *Candida*.

The archetype and most studied member of the SREBP family, the human SREBP1, exhibits dual DNA binding specificity in *in vitro* DNA binding assays: It can bind the palindromic E-box (5’-CANNTG-3’) generally recognized by bHLH proteins as well as a non-palindromic sequence (5’-TCANNCCA-3’) [23]. The protein, however, appears to preferentially bind *in vivo* to the palindromic E-box as revealed by ChIP experiments [25]. Our results indicate that the branch of fungal SREBPs represented by the *C*. *albicans* Tye7 protein shares these same DNA binding features with the human SREBP1. That is, the *C*. *albicans* protein displayed the same dual DNA binding specificity in *in vitro* DNA binding assays (S5C and S5D Fig) and also bound *in vivo* preferentially to a palindromic E-box variant (Fig 2, S8 Fig and [27]). Their similarities in DNA binding profile are in stark contrast to the divergent cellular functions that they govern: While the human SREBP1 regulates the expression of sterol biosynthesis genes, the Tye7 protein controls the expression of sugar acquisition and sugar metabolism genes [27] (Fig 7E). Furthermore, the former harbors the transmembrane domains that are a feature of the family [19] whereas the Tye7 proteins have no traces of any transmembrane domain in their sequences (Fig 1). Thus, the Tye7 branch of fungal SREBPs shares the human SREBP1’s DNA binding features despite the distinct roles that each protein plays in their organisms.

The dual DNA binding ability of the human SREBP1 has been traced back to a tyrosine residue in the DNA binding domain of the protein [23]. Most bHLH proteins have a conserved arginine in this position (instead of the tyrosine) (Fig 1A). The arginine residue forms a stabilizing salt bridge with a conserved glutamate nearby; such structure underlies, at least in part, the protein-DNA contacts with the canonical E-box [24]. This salt bridge cannot be formed when the tyrosine is present, conferring conformational plasticity to accommodate protein-DNA contacts with the non-palindromic sequence (5’-TCANNCCA-3’) besides the palindromic E-box [24]. All the proteins included in our study (Fig 1) harbor the tyrosine residue characteristic of the SREBPs. Yet in contrast to the DNA binding patterns displayed by the *C*. *albicans* Tye7p and human SREBP1, the branch (or branches) of the fungal SREBPs represented by the *C*. *albicans* and *C*. *parapsilosis* Hms1p and the *A*. *fumigatus* SrbAp exhibited a marked preference, both *in vitro* and *in vivo*, for the alternative, non-palindromic sequence (5’-TCANNCCA-3’). These results suggest that the tyrosine residue that is the hallmark of SREBPs enables *alternate* binding specificity in addition to dual DNA binding (the latter is what has been reported in the human SREBP1).

Our DNA binding assays with purified *Ca*Hms1 and *Af*SrbA proteins demonstrate that their preference to bind the non-palindromic sequence (5’-TCANNCCA-3’) over the palindromic E-box is an intrinsic property of the proteins (Fig 4 and S8 Fig). Amino acid residues in the first helix and the loop region of the *Ca*Hms1p were necessary to confer the specificity towards the non-palindromic sequence (Fig 5). Based on crystal structures of various bHLH proteins, the amino acid residues making direct contact with DNA are located within the basic region and first helix of the DNA binding domain [21, 24, 46, 47]: A glutamine and an arginine residues in the first helix and a histidine in the basic region make direct contacts with the bases that comprise the E-box [47]. These three amino acids are fully conserved throughout the fungal SREBPs included in our study (S1 Table). Thus, the changes in DNA binding specificity that we identify in the SREBP family cannot be due to variation in any of these positions. In bHLH regulators such as the *S*. *cerevisiae* Pho4, residues at the boundaries of the loop region (*i*.*e*. towards the end of the first and beginning of the second helices) are known to interact to stabilize the overall structure [47]. We speculate that at least some of the amino acids underlying the change in DNA specificity in Hms1p may, similarly, be involved in “stabilizing” the structure rather than in making direct contacts with DNA. The fact that the first helix and the loop region were necessary imply that more than one single amino acid change is responsible for the switch in specificity. This finding is consistent with the observation that cumulative amino acid changes—which often must occur in a particular order—are usually responsible for the modifications in protein function that occur during evolution [48–50]. Furthermore, these results suggest that “disordered” regions of a protein’s DNA binding domain, such as the loop region in bHLH proteins, may also influence DNA binding specificity.

In contrast to *Ca*Hms1p and *Af*SrbAp, it is apparent that for other SREBPs, such as *Ca*Tye7p and human SREBP1, additional factors may contribute to their *in vivo* DNA binding specificity. We speculate that binding to target promoters with co-factors may be one such determinant. It has been shown, for example, that the human SREBP1 cooperates *in vivo* extensively with the co-factors NFY and SP1 [25]. The *C*. *albicans* Tye7 protein has also been shown to bind to many promoters together with another protein, Gal4 [27]; indeed, both regulators Tye7 and Gal4 are needed to control the expression of glycolysis genes in this species [27, 51].

In *Yarrowia lipolytica*, a species that lies at the very base of the Saccharomycotina, the SREBP *Yl*Sre1 has been shown to be required for switching from yeast to filamentous growth in hypoxia [26]. The data described in this report indicates that two of the three *C*. *albicans* SREBPs regulate the same morphological switch in anaerobic conditions although in the opposite direction: *Ca*Hms1 and *Ca*Cph2 were needed to prevent *Candida* from switching from yeast to filamentous form under these conditions (Fig 7). Thus, the connection of SREBPs to fungal morphology regulation appears to have been maintained throughout the Saccharomycotina evolution. *Ca*Hms1p and *Ca*Cph2p form a regulatory cascade through which the gene encoding the former protein is a direct target of regulation of the latter. Cph2 is the only SREBP in *C*. *albicans* that contains transmembrane domains, a distinctive feature of the family. It is plausible that by being inserted in intracellular membranes, the activity of Cph2p can be modulated by stimuli related to those that regulate the prototypical SREBPs. Hms1p, on the other hand, lacks the transmembrane domains; hence, the activity of this protein most likely responds to different intra- or extra-cellular signals. The Cph2-Hms1 regulatory cascade can, thus, expand the repertoire of stimuli that feed into the circuit to control yeast-to-filament transition [1, 52, 53].

In sum, we have shown that the fungal SREBPs comprise several branches that differ from one another in their DNA binding preferences and in the biological processes that they regulate. A key element in the diversification of the family appears to be the intrinsic structure of the DNA binding domain of the SREBPs which allows these proteins to adopt two distinct conformations and therefore recognize at least two different DNA sequences. Our findings suggest that this promiscuous state was resolved during evolution of the family: One branch tilted the preference towards one of the DNA motifs largely through amino acid changes in the same protein whereas another branch tilted the preference towards the second DNA motif. We posit that the diversification in their DNA binding preferences enabled the SREBPs to expand and regulate diverse cellular processes in fungi.

## Materials and methods

### SREBP nomenclature

The standard and systematic names of the main SREBP genes included in this study are as follows: *TYE7* (*ORF19*.*4941* or *C1_13140C_A* in *C*. *albicans*; *YOR344C* in *S*. *cerevisiae*); *CPH2* (*ORF19*.*1187* or *C6_00280W_A* in *C*. *albicans*; *YOR032C* in *S*. *cerevisiae*); *HMS1* (*ORF19*.*921* or *C5_00670C_A* in *C*. *albicans*). Notice that although the standard name of the *S*. *cerevisiae* gene *YOR032C* in the *Saccharomyces* genome database is *HMS1*, our phylogenetic reconstruction places it closer to the *C*. *albicans CPH2* branch (Fig 1B).

### *C*. *albicans* strains

All *C*. *albicans* strains used in this study are listed in S4 Table and are derivatives of the clinical isolate SC5314 [54]. For the construction of the epitope-tagged strain Cph2-MYC, which was used in ChIP experiments, a DNA fragment encoding 13× MYC followed by the *SAT1*/flipper cassette was amplified by PCR from plasmid pADH34 [55] with oligos described in S5 Table and integrated in the *CPH2* (*ORF19*.*1187*) locus. This construct effectively truncates one of the *CPH2* alleles at codon 407 and inserts the MYC tag at this position. The *SAT1* cassette was then removed as described [56]. DNA sequencing and Western blot analysis confirmed the correct insertion of the tag and the expression of the tagged protein at the expected size, respectively.

### Plasmid construction

All plasmids used for recombinant protein expression are listed in S6 Table. The putative DNA binding domains of *Ca*Cph2 (amino acids 197–302), *Ca*Hms1 (amino acids 463–686), *Ca*Tye7 (amino acids 121–269), *Cp*Hms1 (amino acids 486–659) and *Af*SrbA (amino acids 145–266) were amplified from genomic DNA of each species and introduced into plasmids pLIC-H3 [57] and pbRZ75 [58] (both derivatives of pET28b). These plasmids were designed to produce recombinant N-terminal 6×His or 6×His-MBP (maltose binding protein) tagged proteins.

Chimeric proteins were constructed by (1) replacing residues 211–232 from *Ca*Cph2 by residues 489–510 from *Ca*Hms1 to generate chimeric helix 1 protein; (2) replacing residues 255–281 from *Ca*Cph2 by residues 625–651 from *Ca*Hms1 to generate chimeric helix 2 protein; (3) replacing residues 211–255 from *Ca*Cph2 by residues 489–629 from *Ca*Hms1 to generate chimeric helix1-loop protein; and (4) replacing the loop of *Ca*Cph2 (236–255) by the loop of *Ca*Hms1 (506–625) to generate the chimeric loop protein.

The DNA fragments encoding the reconstructed ancestral proteins were generated by gene synthesis (Invitrogen GeneArt Gene Synthesis). These fragments included restriction sites for cloning into pLIC-H3 [57].

### Recombinant protein purification

*E*. *coli* BL21 was used as the host of the expression plasmids. For recombinant protein overexpression, bacterial cells were grown to an OD_600_ of approximately 0.8 and induced with 0.5 mM IPTG. Cultures were grown further for 3 hours, pelleted and stored at -80°C. Cells were lysed by sonication. His-tagged proteins were affinity purified from the lysate using Ni-NTA agarose beads (Qiagen). Amicon Ultra-15 centrifugal filters (Merck) (10 or 30K membranes depending on protein size) were used to exchange buffer and concentrate the proteins. Protein concentration was estimated in Rothi‐blue (Carl Roth, Germany) stained gels using known amounts of bovine serum albumin as standards.

### Electrophoretic mobility gel shift assays

EMSAs were carried out as described [58]. Competition assays with unlabeled DNAs were performed as reported [13]. Gel shift assays with fluorescently-labeled DNA sequences (shown in S4 Fig) were conducted in a similar fashion to those with radiolabeled DNA except that larger amounts of Cy5-labeled non-palindromic and Cy3-labeled palindromic DNA fragments, alone or together, were incubated with a fixed amount of protein.

### MITOMI 2.0

MITOMI experiments were carried out essentially as described [13, 32]. Briefly, the DNA binding domains of *C*. *albicans* Hms1p (amino acids 463–685), Cph2p (amino acids 197–302) and Tye7p (amino acids 159–269) tagged with GFP were generated with an *in vitro* transcription-translation system (Promega) and added to a microfluidics device containing the Cy5-labeled DNA library. All experiments used a 740-oligonucleotide pseudorandom DNA library containing all possible 8-nucleotide sequences (S7 Table). The library was designed to minimize similarities between k-mers represented on a given strand and thereby reduce the chance of multiple binding sites. Protein-DNA interactions were trapped at equilibrium. After a series of washings where unbound DNA and proteins were washed out, the GFP/Cy5 intensity ratio was measured in every chamber of the device. Experiments were performed in duplicates. Cytoscape (v3.4) [59] was used to visualize the data. MatrixREDUCE [33] was used to search overrepresented DNA motifs. The model variants (topologies) X6, X7, X3N2X3 and X4N2X3 (in forward, reverse or both strands), as implemented in MatrixREDUCE, were employed to query the datasets. The DNA motifs with *P* < 1 × 10^−10^ were ranked according to their *r*^*2*^ and *P*-values, which were calculated by the same software.

### Phylogenetic reconstruction

Fungal protein sequences were retrieved from UniProt [60]. The maximum likelihood tree was constructed by aligning the basic region, first helix and second helix of the DNA binding domain of 198 fungal SREBPs in MEGA7 [61]. Due to its variability in sequence and length, only the last five residues of the loop were taken into account, the rest of the loop region was omitted from the alignment. Only proteins carrying the characteristic tyrosine residue in the first helix (a defining feature of the SREBP family) were included in the analysis. ProtTest [62] was used to find the best-fit model to infer the phylogenetic tree (LG+G). The presence of transmembrane domains was predicted with *OCTOPUS* [63].

### Ancestral protein reconstruction

Phylobot [37] was used to infer ancestral protein sequences at specific nodes of the phylogenetic tree. The model used to infer the tree was PROTGAMMALG (tested for best-fit model with ProtTest) for all cases. The reconstructed protein sequence for Anc4 exhibited low levels of uncertainty. For Anc5, due to higher levels of sequence uncertainty, we carried out two reconstructions: (1) Using Pho4 as the only outside sequence; and (2) including mouse and human SREBP sequences in addition to Pho4 as outside sequences. Two alignment models, MUSCLE and msaprobs, were considered. Eight versions of Anc5, which differed from one another at residues in the first helix, were synthesized and their overall ability to bind to DNA was evaluated by EMSAs. Due to the high variability in the loop segment, this particular portion of the DNA binding domain could be neither properly aligned nor reconstructed. Given its short size (55 amino acid residues), the corresponding amino acid sequence of *Ca*Cph2p was used to fill the loop segment in all ancestral proteins.

### Full-genome chromatin immunoprecipitation

MYC-tagged and untagged *C*. *albicans* strains (the latter served as a negative control) were grown in YPD broth at 30°C until mid-log phase. ChIP was carried out as described [55] with the following modifications: Input and immunoprecipitated DNAs were directly used to generate libraries for sequencing with the NEBNext ChIP-Seq Library Prep Master Mix Set for Illumina (New England Biosciences). DNA sequencing was carried out by GATC Biotech (Konstanz, Germany) using standard procedures. The reads were aligned to the *C*. *albicans* genome using Bowtie2 [64] with default parameters. Between 3–10 million reads per sample were uniquely aligned to the genome. Peak calling and visualization were performed with MACS2 [65] (using default parameters) and MochiView [66], respectively. To ensure the generation of a high confidence dataset, in addition to the standard computational analyses we manually curated all the extracted peaks using the following criteria: (1) Peaks that appeared in both the untagged control and the Cph2-MYC tagged strain were removed; (2) peaks located within annotated ORFs were ignored; (3) peaks located around highly expressed genes (particularly ribosomal genes) were also discarded because based on previous experience (*e*.*g*. [29, 35, 67, 68]) these places tend to bind to almost all DNA binding proteins non-specifically; and (4) only peaks that appeared significant in the MACS2 analysis in at least two of three replicates were taken into account. Motif finding analysis was performed with MochiView by providing 500 nt DNA sequences surrounding the high-confidence peaks using the software’s default parameters.

### Transcriptome analysis

*C*. *albicans* reference strain, *hms1* and *cph2* deletion mutants were grown in Todd-Hewitt broth in an anaerobic chamber at 37°C for 24 hours. The culture medium had been placed in the anaerobic chamber at least two days before inoculation to remove any oxygen traces. Two independent replicates were used for the analysis. Total RNA extraction and cDNA synthesis was performed as described [69]. Quality control, mapping and differential gene expression was carried out as reported [45]. We obtained 63–91 million reads per sample which were then aligned to the *C*. *albicans* genome using STAR v2.5.2b [70] with default parameters (>97% of reads of each sample were uniquely aligned to the *C*. *albicans* genome). Read counts were loaded into R (v3.3.2) and analyzed with the DESeq2 [71] package (v1.14.1). With our depth of sequencing, significant numbers of reads were detected for ~6,100 annotated ORFs. Cytoscape [59] (v3.4) was used to visualize the data and generate the network graphs.

### Cell morphology determination

Overnight *C*. *albicans* cultures (in Todd-Hewitt broth at 30°C) were diluted to OD_600_ ~0.1 in fresh Todd-Hewitt broth and incubated at 37°C under anaerobic conditions for 24 hours. The medium used to dilute the overnight culture had been pre-incubated in an anaerobic chamber for 48 hours to achieve complete anaerobiosis. After the 24-hour period of growth, cells were washed with sterile PBS and fixed in glass slides for morphology evaluation under the microscope.

### Reverse transcription and real-time PCR

Reference strain, *cph2* and *efg1* deletion mutants were grown under anaerobic conditions as described above. Total RNA purification and cDNA synthesis were performed as described [69]. Real time PCR was used to quantify specific transcripts (oligos listed in S5 Table). The experimentally validated *TAF10* transcript [72] served as a reference control for the qPCR.

### Statistical analyses

The significance of the overlap between the differentially expressed genes from our RNA-seq datasets was estimated using the hypergeometric test. The Gene Ontology Term Finder of the Candida Genome Database (www.candidagenome.org) was used to identify enriched processes in our RNA-seq dataset. The student *t*-test for unpaired samples was used to assess statistical differences between transcript levels.

### Accession numbers

The ChIP-Seq and RNA-Seq data reported in this article have been deposited in the NCBI Gene Expression Omnibus (GEO) database under accession numbers GSE118419, GSE118416 (ChIP-Seq) and GSE118414 (RNA-Seq).

## Supporting information

S1 Table SREBP sequences and DNA binding domain alignment. This file contains the protein sequences of all fungal SREBPs included in the study and the sequence alignment of their DNA binding domain. Only the non-redundant sequences (N = 114) are displayed in the alignment.
(XLSX)

S2 Table Comprehensive list of DNA motifs derived from MITOMI data. This file includes all the DNA motifs resulting from the analysis of the MITOMI data with MatrixREDUCE (*P* < 1 × 10^−10^). The derived motifs are ranked according to *r*^*2*^ and *P*-values. The model variants (topologies) X6, X7, X3N2X3 and X4N2X3 (in forward, reverse or both strands) were employed to query the datasets. Top-scoring motifs are highlighted in blue. Representative motifs displayed in Fig 2 are highlighted in green.
(XLSX)

S3 Table List of differentially expressed transcripts in the *cph2* and *hms1* deletion mutant strains. This file includes a list of the *C*. *albicans* genes whose expression is dependent on *HMS1* and/or *CPH2* under anaerobic conditions (log_2_ fold-change values > |1| and negative log_10_ (*P*-value) > 10). The file also contains the log_2_ fold-change values (mutant/wt) for all annotated transcripts in the *C*. *albicans* genome.
(XLSX)

S4 Table *Candida albicans* strains used in this study. (PDF)

S5 Table Oligonucleotides used in this study. (PDF)

S6 Table Recombinant protein expression plasmids. (PDF)

S7 Table Library of 740 oligonucleotides used in MITOMI. (XLSX)

S1 Fig Extended phylogenetic tree of fungal SREBPs. Reconstruction was carried out as described in Fig 1B. Blue dots indicate the presence or absence of transmembrane domains. The three *C*. *albicans* SREBPs, Cph2 (orange), Hms1 (cyan) and Tye7 (red) are highlighted.
(PDF)

S2 Fig Distribution of top 30% (*A*) and top 50% (*B*) oligonucleotides bound by each protein in MITOMI experiments. Each purple dot represents one oligonucleotide. Distances between proteins (cyan, orange and red circles) are inversely proportional to their degree of binding overlap.
(PDF)

S3 Fig DNA binding profile by *Ca*Cph2p. (*A*) Schematic representation of the *Ca*Cph2-MYC construct used for ChIP. (*B*) List of DNA regions occupied by *Ca*Cph2p based on our ChIP-Seq experiment. The DNA motif derived from the ChIP data was used to calculate motif scores at peak locations. (*C*) Gel shit assay probing the binding of the purified *Ca*Cph2 protein (0, 0.0012, 0.006, 0.02 and 0.1 nM) to the indicated P^32^-labeled DNA fragment (taken from the upstream intergenic region of *ORF19*.*921*) which harbors an instance of the putative Cph2 motif (in black). DNA binding is strongly reduced when point mutations (in red) are introduced in the binding site.
(PDF)

S4 Fig Gel shift assay with fluorescently-labeled DNA sequences competing for the same pool of *Ca*Hms1 protein. Increasing amounts (0.56, 1.67, 5, 15 and 45 ng) of Cy5-labeled non-palindromic and Cy3-labeled palindromic DNA fragments, alone or together, were incubated with purified Hms1 protein, and resolved in 6% polyacrylamide gels run with 0.5× TGE. The images of the gels taken in the Cy5 and Cy3 channels are shown at the top and bottom, respectively. Notice the strong preference (>10-fold) of the protein for the Cy5-labeled non-palindromic site.
(PDF)

S5 Fig Gel shift competition assays with the purified DNA binding domain of *Ca*Cph2p (*A*) and *Ca*Tye7p (*C*). The P32-radiolabeled DNA (and the unlabeled competitors) contained either the non-palindromic binding site (top panels) or a palindromic E-box sequence (bottom panels). (*B* and *D*) Quantification of competition assays; best-fit curves are included.
(PDF)

S6 Fig Ancestral protein reconstruction and synthesis. (*A*) Phylobot output displaying the probabilities of the given residues at each position of the reconstructed ancestors. Three reconstructions applying different alignment methods were used to infer the sequence of the “oldest” ancestor (Anc5). (*B*) Amino acid sequences of eight different versions of Anc5 that were synthesized and tested in *in vitro* gel shift experiments. Anc5.3 showed the highest affinity for DNA; hence it was selected for further characterization. Dots in the alignment represent the same amino acid residue written at the top of the column. (*C*) Gel shift assays testing the binding specificity of the purified Anc5 protein (0, 6.25, 25, 100 and 400 nM) towards the palindromic (left) and the non-palindromic (right) DNA sequences. Point mutations (in red) introduced in the DNA fragment abolished binding.
(PDF)

S7 Fig Co-regulation of transcripts by *CaHMS1* and *CaCPH2*. Shown is the set of genes co-regulated by the two proteins at different thresholds. A stringent threshold (log_2_ fold change > |2| and -log_10_ *P* value > 20) was applied in (*A*); a less stringent threshold (log_2_ fold change > |1| and *P* value < 0.001) is applied in (*B*). Up-regulated genes are shown in yellow and down-regulated genes in blue. The hypergeometric distribution was employed to calculate the significance of the overlap.
(PDF)

S8 Fig Gel shift competition assays with DNA fragments varying exclusively in two nucleotides within the core binding sequence. (*A*) Full sequences of the two DNA fragments evaluated. (*B* and *C*) Gel shift competition assays for the *Ca*Hms1 (*B*) and *Ca*Tye7 (*C*) proteins. Assays were carried out as described in the legend to Fig 4B. Quantification of competition assays is shown to the right; best-fit curves are included. Notice that the two proteins show opposing DNA binding preferences.
(PDF)
