# Supplementary material for: Diversification of DNA binding specificities enabled SREBP transcription regulators to expand the repertoire of cellular functions that they govern in fungi
Source: PLoS Genet. 2018 Dec 31;14(12):e1007884. doi: 10.1371/journal.pgen.1007884 (PMC6329520; doi:10.1371/journal.pgen.1007884)
Supplement: S4 Table — (PDF) [file pgen.1007884.s004.pdf]

S4 Table. *C. albicans* strains used in this study

Strain	Genotype	Source
SN250	$\frac{ura3\Delta::\lambda imm434::URA3-IRO1}{ura3\Delta::\lambda imm434}\ \frac{arg4::hisG}{arg4::hisG}\ \frac{his1::hisG}{his1::hisG}\ \frac{leu2::hisG::CdHIS1}{leu2::hisG::CmLEU2}$	[1]
TF02	$\frac{ura3\Delta::\lambda imm434::URA3-IRO1}{ura3\Delta::\lambda imm434}\ \frac{arg4::hisG}{arg4::hisG}\ \frac{his1::hisG}{his1::hisG}\ \frac{hms1\Delta::CdHIS1}{hms1\Delta::CmLEU2}$	[1]
TF138	$\frac{ura3\Delta::\lambda imm434::URA3-IRO1}{ura3\Delta::\lambda imm434}\ \frac{arg4::hisG}{arg4::hisG}\ \frac{his1::hisG}{his1::hisG}\ \frac{cph2\Delta::CdHIS1}{cph2\Delta::CmLEU2}$	[1]
JCP880	$\frac{ura3\Delta::\lambda imm434::URA3-IRO1}{ura3\Delta::\lambda imm434}\ \frac{arg4::hisG}{arg4::hisG}\ \frac{his1::hisG}{his1::hisG}\ \frac{leu2::hisG::CdHIS1}{leu2::hisG::CmLEU2}\ \frac{CPH2\ (1-407)-myc}{CPH2}$	This study

1. Noble SM, Johnson AD. Strains and strategies for large-scale gene deletion studies of the diploid human fungal pathogen *Candida albicans*. Eukaryot Cell. 2005;4: 298–309.
