# Supplementary material for: Diversification of DNA binding specificities enabled SREBP transcription regulators to expand the repertoire of cellular functions that they govern in fungi
Source: PLoS Genet. 2018 Dec 31;14(12):e1007884. doi: 10.1371/journal.pgen.1007884 (PMC6329520; doi:10.1371/journal.pgen.1007884)
Supplement: S5 Table — (PDF) [file pgen.1007884.s005.pdf]

S5 Table. Oligos used in this study

Number	Description	Sequence (5' to 3')
Oligos used for plasmid construction		
1425	*Ca*Hms1 into pLIC-H3 FW	AGCAGCCCCGGGTCAACAATTATTAATGAACCAAC
1429	*Ca*Hms1 into pLIC-H3 RV	GTGGTGCTCGAGCTACATATCTTCATCAATAACTAAAC
1426	*Ca*Cph2 into pLIC-H3 FW	AGCAGCCCCGGGGCCAAAGTCACAAAGCCAAAATC
1430	*Ca*Cph2 into pLIC-H3 RV	GTGGTGCTCGAGTTATAATGACTTTGGATTCATGTTGGC
1513	*Ca*Tye7 into pbRZ75 FW	CATATGGCTAGCACTAAACCAAAGAGAAGAGCACC
1660	*Ca*Tye7 into pbRZ75 RV	GTGGTGCTCGAGCTATATTTCACCACCCAATTTAATAAC
2044	*Cp*Hms1 into pbRZ75 FW 1A	CATATGGCTAGCAGTACAACAATTACAGCTTCCAGTG
2006	*Cp*Hms1 into pbRZ75 RV 1B	GATGGAATGGATTTGTCGATTCTTGATGATAAG
2007	*Cp*Hms1 into pbRZ75 FW 2A	CTTATCATCAAGAATCGACAAATCCATTCCATC
2008	*Cp*Hms1 into pbRZ75 RV 2B	GTGGTGCTCGAGTTATTCTTCTTCATCAAAATCAACACCC
2030	*Af*SrbA into pLIC-H3 FW	AGCAGCCCCGGGACTGGAAGCGATGACGATGGATCTA
2031	*Af*SrbA into pLIC-H3 RV	GTGGTGCTCGAGCTAGGTAACAATCTGATCAGCGGCCTTG
1783	Helix1 chimera into pLIC-H3 1A	ATTAAATCAGCTCATAATGTAATTGAACAACGATATCGAAATAAAATTAATGATAAATTTAATGCTTTACAAAATTCT
1784	Helix1 chimera into pLIC-H3 1B	AGAATTTTGTAAAGCATTAAATTTATCATTAATTTTATTTCGATATCGTTGTTCAATTACATTATGAGCTGATTTAAT
1357	Helix1 chimera into pLIC-H3 2A	GCACCATCATCATCACCATC
1785	Helix1 chimera into pLIC-H3 2B	CAATTACATTATGAGCTGATTTAATTTTTGATTTTGGC
1786	Helix1 chimera into pLIC-H3 3A	TAAATTTAATGCTTTACAAAATTCTGTGCCTGCTC
1358	Helix1 chimera into pLIC-H3 3B	CTTTCGGGCTTTGTTAGCAG
1839	Helix 2 chimera into pLIC-H3 1B	TTGTTCCTTTATTCAATTTTCTTGCTGGTGTTAACC
1840	Helix 2 chimera into pLIC-H3 2A	GCAAGAAAATTGAATAAAGGAACAATATTAGCTAAATCTATTGAATATATTAAATTTTTAGAAATGAAAAATGAAAGA
1841	Helix 2 chimera into pLIC-H3 2B	TCTTTCATTTTTCATTTCTAAAAATTTAATATATTCAATAGATTTAGCTAATATTGTTCCTTTATTCAATTTTCTTGC
1862	Helix 2 chimera into pLIC-H3 3A	ATTTTTAGAAATGAAAAATGAAAGATTGAAACAACA
1843	Helix1+Loop chimera 1B	GCTTTTTTTGGGCTAATATTCTTAAAGCAGGCACA
1844	Helix1+Loop chimera 2A	TTAAGAATATTAGCCCAAAA
1845	Helix1+Loop chimera 2B	TGGTTCTAATCCTTCTAAAT
1863	Helix1+Loop chimera 3A	TATTGATTTAGAAGGATTAGAACCAGCATCTAAGTTAAAC
2071	Loop chimera 1B	GCTTTTTTTGGGCTAATATTCTTAAAGCAGGCACAG
1844	Loop chimera 2A	TTAAGAATATTAGCCCAAAA
1949	Loop chimera 2B	ATTCAATTTTCTTGCTGGTTC
2000	Loop chimera 3A	ATTAGAACCAGCAAGAAAATTGAATAAAGCTAGTGTTTTAACC
1358	Loop chimera 3B	CTTTCGGGCTTTGTTAGCAG
Oligos used for MYC tagging		
1432	Cph2-MYC FW	TGCCTTGCCATTTTCGTATTTGTTTCCTAATGCTATTCTTAACCCATCGCCATTGACTATTCAATTACGGATCCCCGGGTTAATTAACGG
1693	Cph2-MYC RV	AACCCACATTCCTTATGAAACAAAATAAAATTAAACAATCTATACCTGAAAAAAAGAAACACTGGCGGCCGCTCTAGAACTAGTGGATC
Oligos used for qPCR		
2261	*ORF19.3242* FW	GCGGAGACAAAGATCGACAC
2262	*ORF19.3242* RV	TATTACTGACGCTGGTGGGG
1829	*ORF19.921* FW	GACACCAATCTCATCAATTAC

1830	*ORF19.921* RV	AAATCTACTGGGAATCCTCC
2462	*ORF19.4941* FW	CAAACCACAAGCAACTCCGA
2463	*ORF19.4941* RV	TGCGCAATACTGTCTGATGTG
