# Supplementary material for: Diversification of DNA binding specificities enabled SREBP transcription regulators to expand the repertoire of cellular functions that they govern in fungi
Source: PLoS Genet. 2018 Dec 31;14(12):e1007884. doi: 10.1371/journal.pgen.1007884 (PMC6329520; doi:10.1371/journal.pgen.1007884)
Supplement: S6 Table — (PDF) [file pgen.1007884.s006.pdf]

S6 Table. Recombinant protein expression plasmids

Plasmid name	Plasmid number	Description	Reference
His6-*Ca*Cph2	JCP567	pLIC-H3 with 6xHis *Ca*Cph2 (aa197-302)	This study
His6-MBP-*Ca*Tye7	JCP726	pbRZ75 with 6xHis-MBP *Ca*Tye7 (aa121-269)	This study
His6-*Ca*Hms1	JCP565	pLIC-H3 with 6xHis *Ca*Hms1 (aa463-686)	This study
His6-MBP-*Cp*Hms1	JCP833	pbRZ75 with 6xHis-MBP *Cp*Hms1 (aa486-659)	This study
His6-*Af*SrbA	JCP831	pLIC-H3 with 6xHis *Af*SrbA (aa463-686)	This study
His6-Chimera1	JCP822	pLIC-H3 with 6xHis Helix1 chimera	This study
His6-Chimera2	JCP823	pLIC-H3 with 6xHis Helix2 chimera	This study
His6-Chimera3	JCP824	pLIC-H3 with 6xHis Helix1 + Loop chimera	This study
His6-Chimera4	JCP836	pLIC-H3 with 6xHis Loop chimera	This study
His6-Anc4	JCP817	pLIC-H3 with 6xHis Ancestor 4	This study
His6-Anc5.3	JCP921	pLIC-H3 with 6xHis Ancestor 5.3	This study
