# Supplementary figures and images for: Diversification of DNA binding specificities enabled SREBP transcription regulators to expand the repertoire of cellular functions that they govern in fungi
Source: PLoS Genet. 2018 Dec 31;14(12):e1007884. doi: 10.1371/journal.pgen.1007884 (PMC6329520; doi:10.1371/journal.pgen.1007884)

S1 Fig.

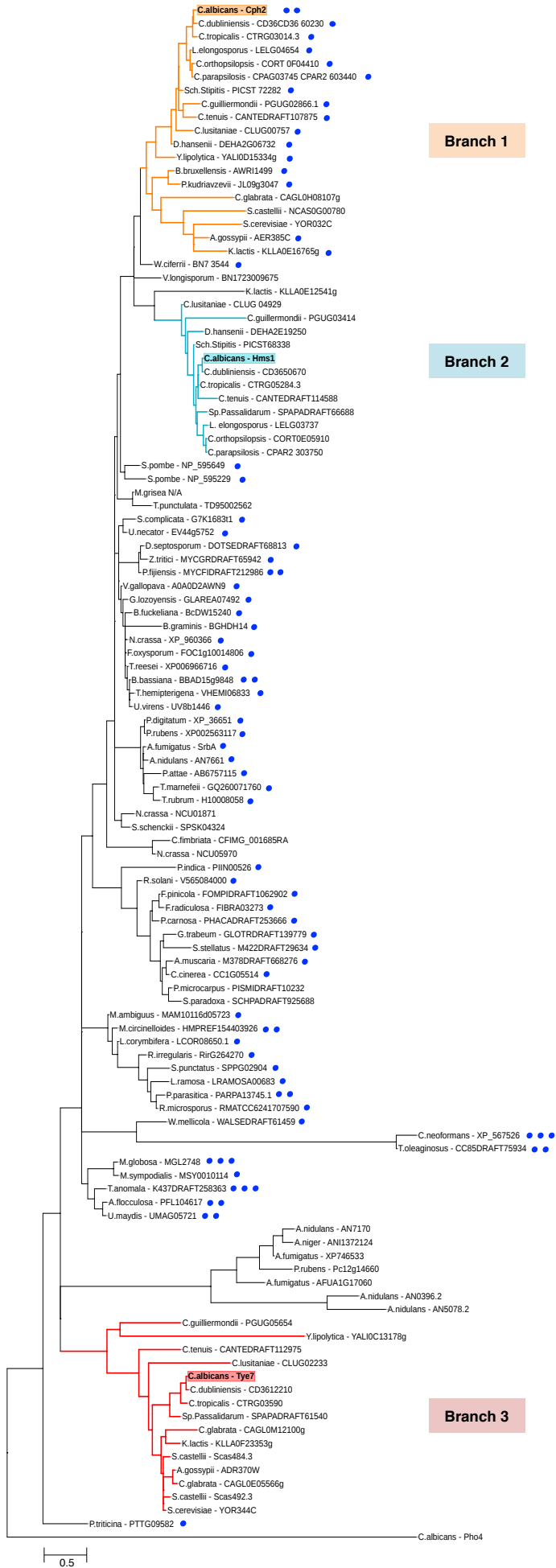

Supplement: S1 Fig — Reconstruction was carried out as described in Fig 1B. Blue dots indicate the presence or absence of transmembrane domains. The three C. albicans SREBPs, Cph2 (orange), Hms1 (cyan) and Tye7 (red) are highlighted. (PDF) [file pgen.1007884.s008.pdf]

S2 Fig.

A

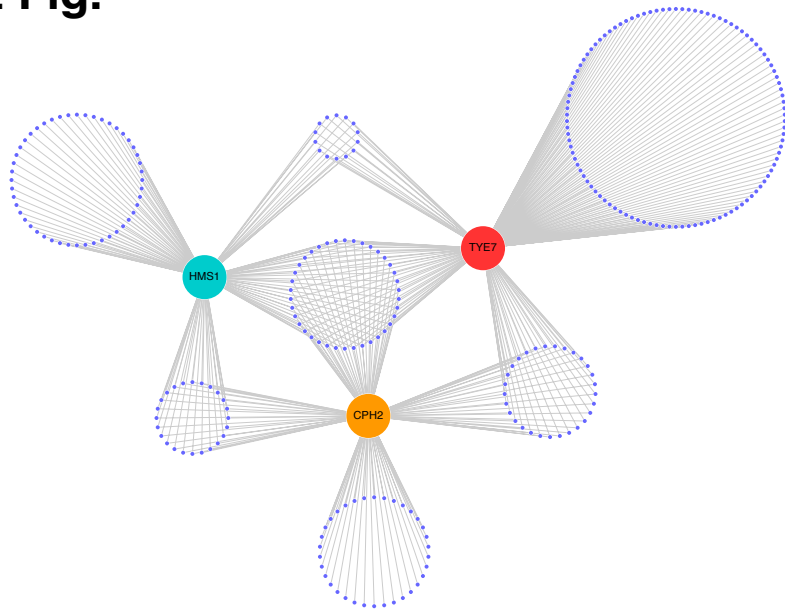

B

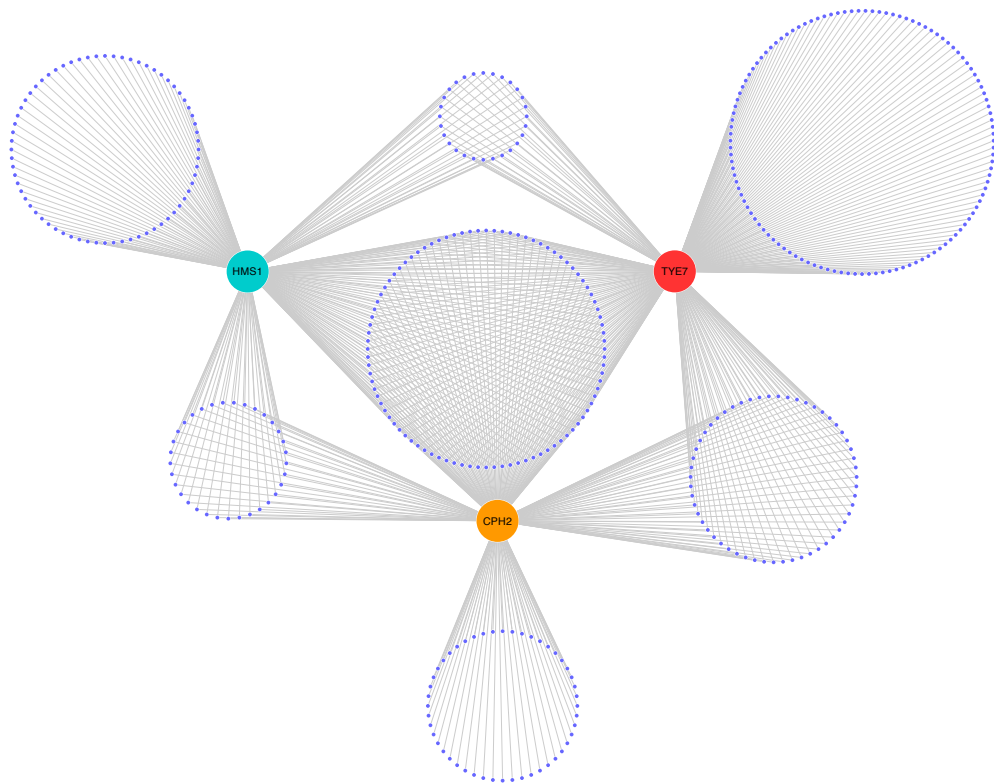

Supplement: S2 Fig — Distribution of top 30% (A) and top 50% (B) oligonucleotides bound by each protein in MITOMI experiments. Each purple dot represents one oligonucleotide. Distances between proteins (cyan, orange and red circles) are inversely proportional to their degree of binding overlap. (PDF) [file pgen.1007884.s009.pdf]

**S4 Fig.**

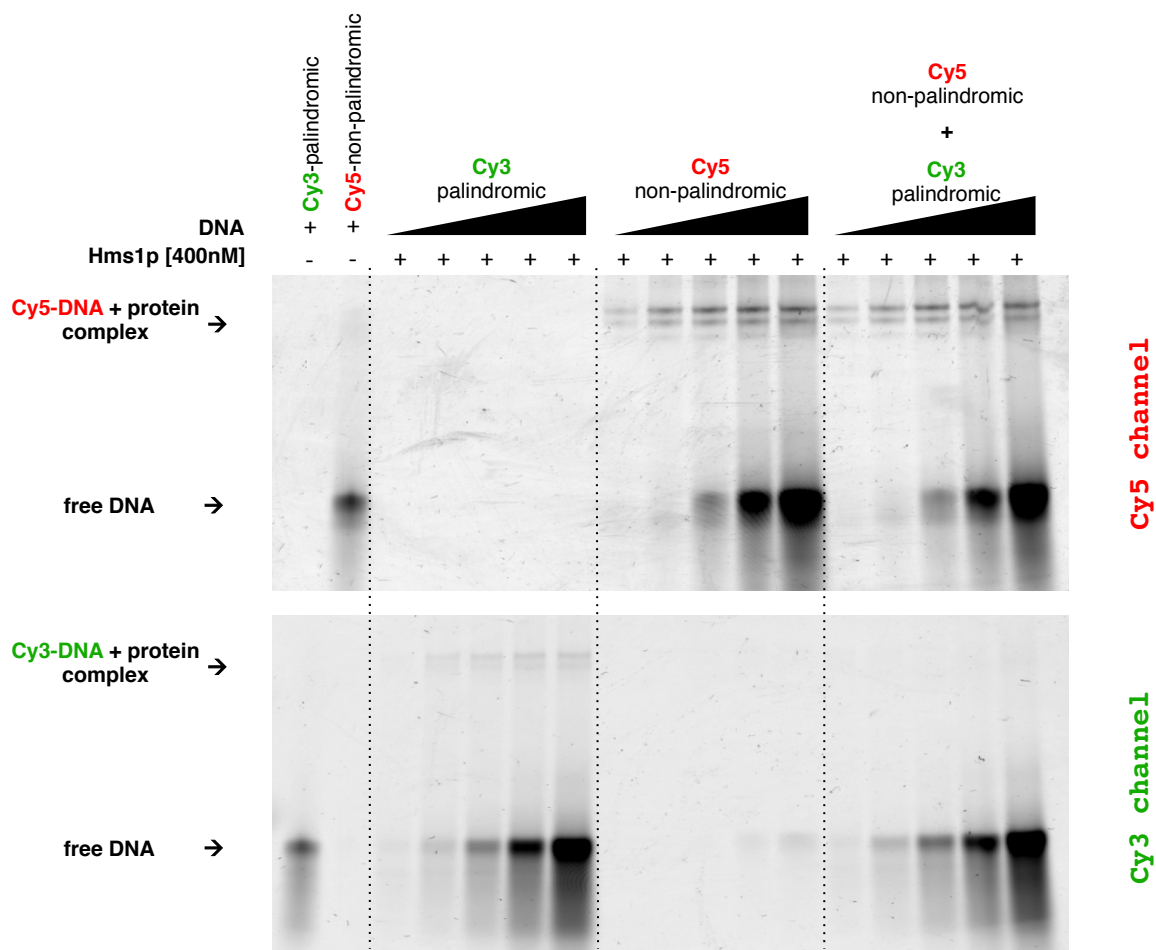

Supplement: S4 Fig — Increasing amounts (0.56, 1.67, 5, 15 and 45 ng) of Cy5-labeled non-palindromic and Cy3-labeled palindromic DNA fragments, alone or together, were incubated with purified Hms1 protein, and resolved in 6% polyacrylamide gels run with 0.5× TGE. The images of the gels taken in the Cy5 and Cy3 channels are shown at the top and bottom, respectively. Notice the strong preference (>10-fold) of the protein for the Cy5-labeled non-palindromic site. (PDF) [file pgen.1007884.s011.pdf]

S5 Fig.

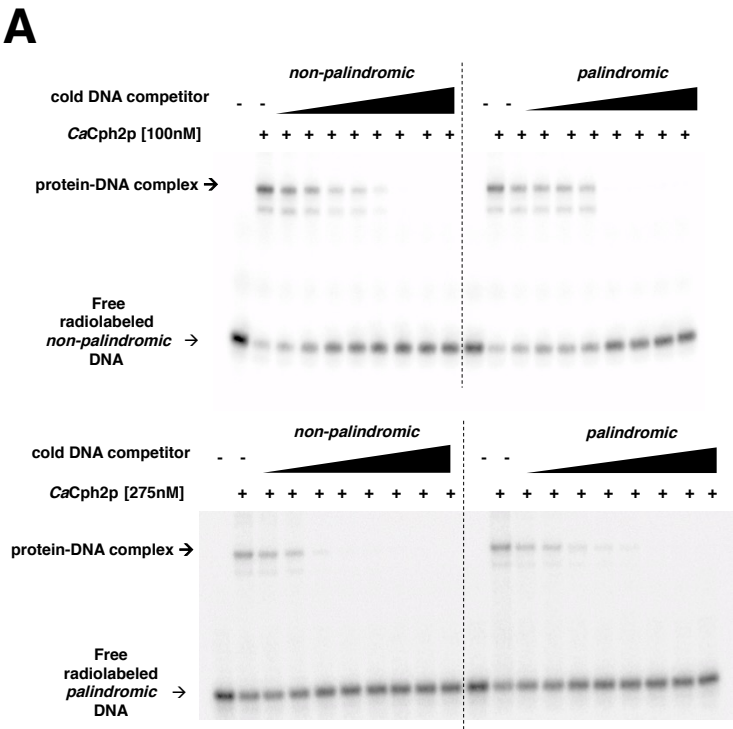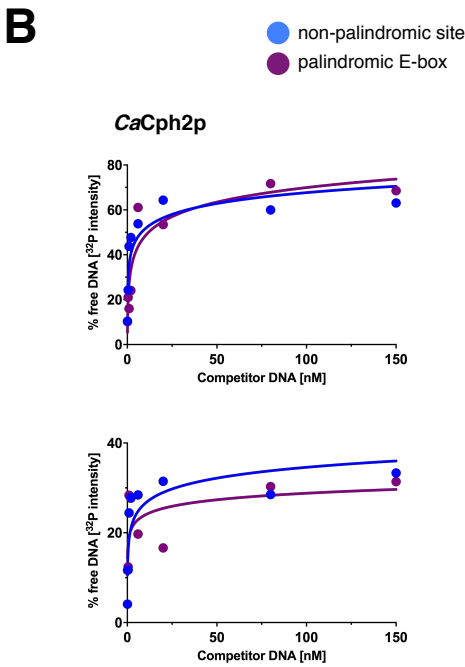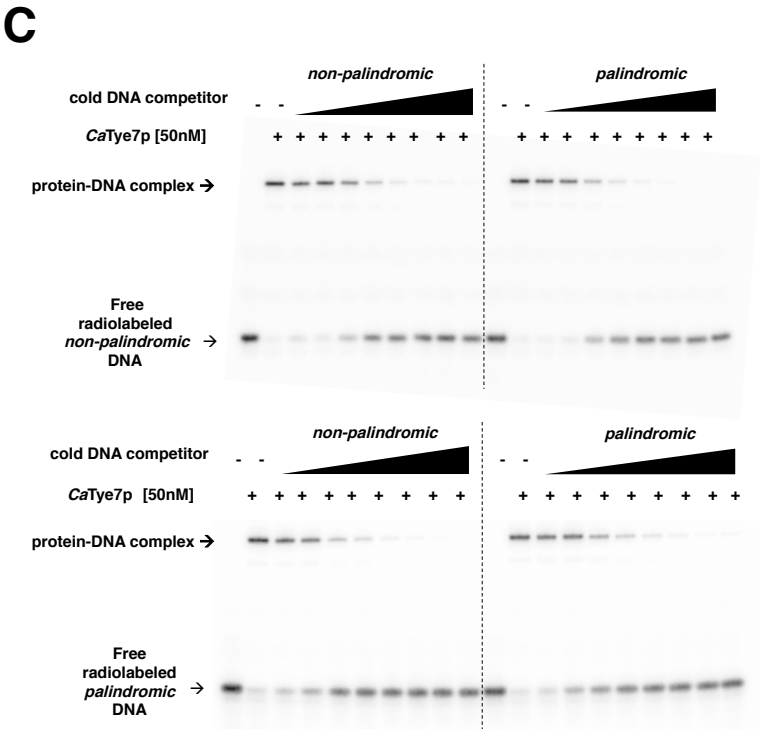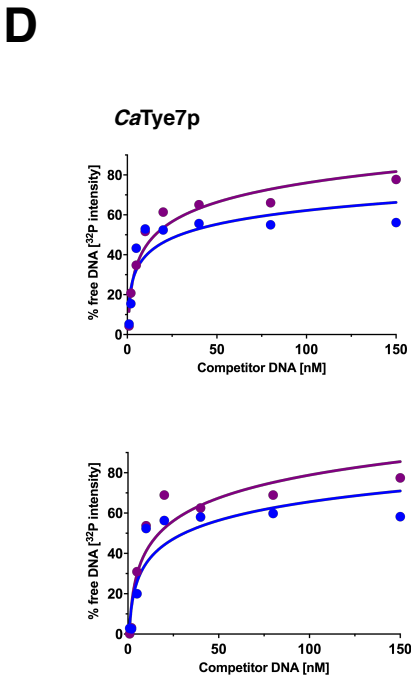

Supplement: S5 Fig — Gel shift competition assays with the purified DNA binding domain of CaCph2p (A) and CaTye7p (C). The P32-radiolabeled DNA (and the unlabeled competitors) contained either the non-palindromic binding site (top panels) or a palindromic E-box sequence (bottom panels). (B and D) Quantification of competition assays; best-fit curves are included. (PDF) [file pgen.1007884.s012.pdf]

S7 Fig.

A

Threshold:  
log2 Fold change |2|  
-log10 *P*-value >20

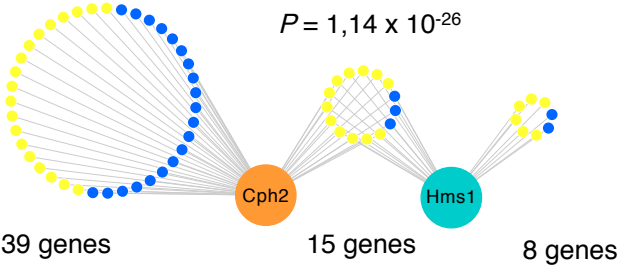

B

Threshold:  
log2 Fold change >1  
*P*-value < 0,001

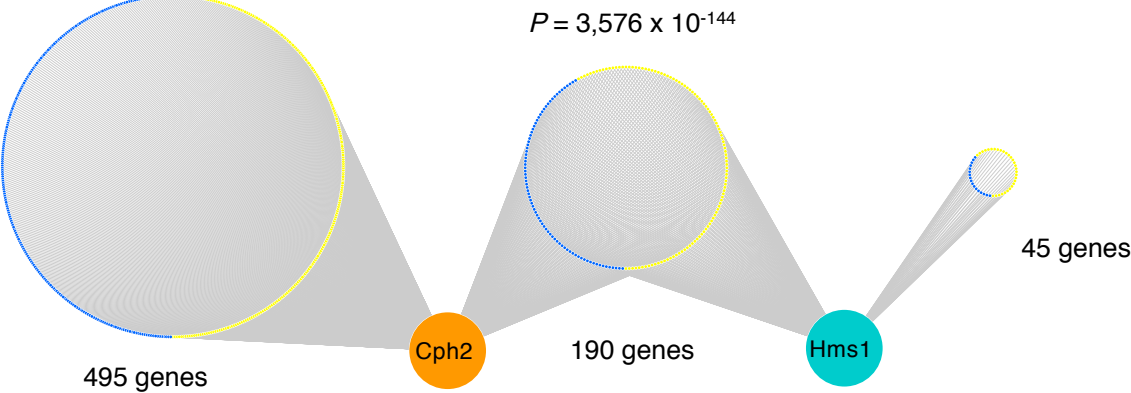

Supplement: S7 Fig — Shown is the set of genes co-regulated by the two proteins at different thresholds. A stringent threshold (log2 fold change > |2| and -log10 P value > 20) was applied in (A); a less stringent threshold (log2 fold change > |1| and P value < 0.001) is applied in (B). Up-regulated genes are shown in yellow and down-regulated genes in blue. The hypergeometric distribution was employed to calculate the significance of the overlap. (PDF) [file pgen.1007884.s014.pdf]

S8 Fig.

A

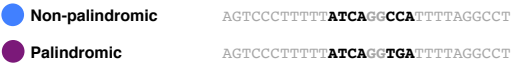

B

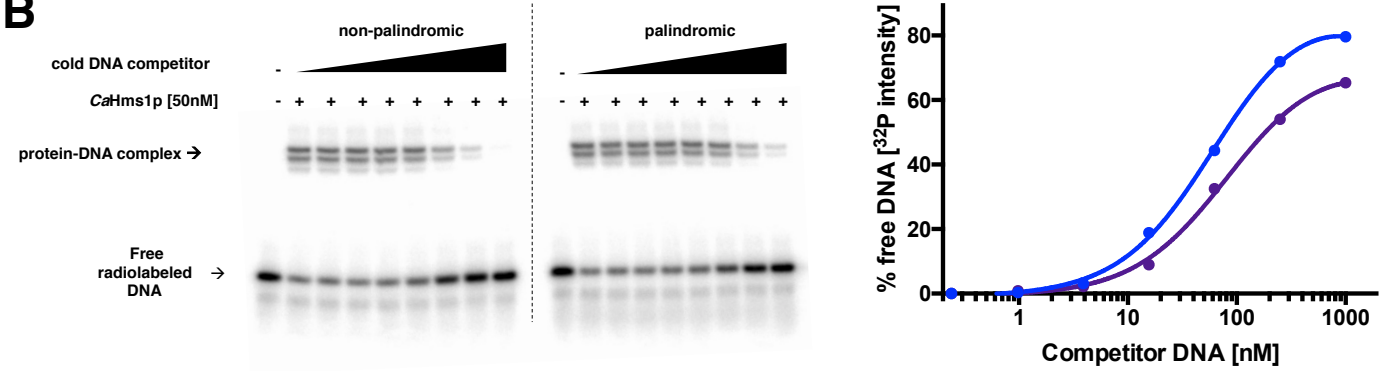

C

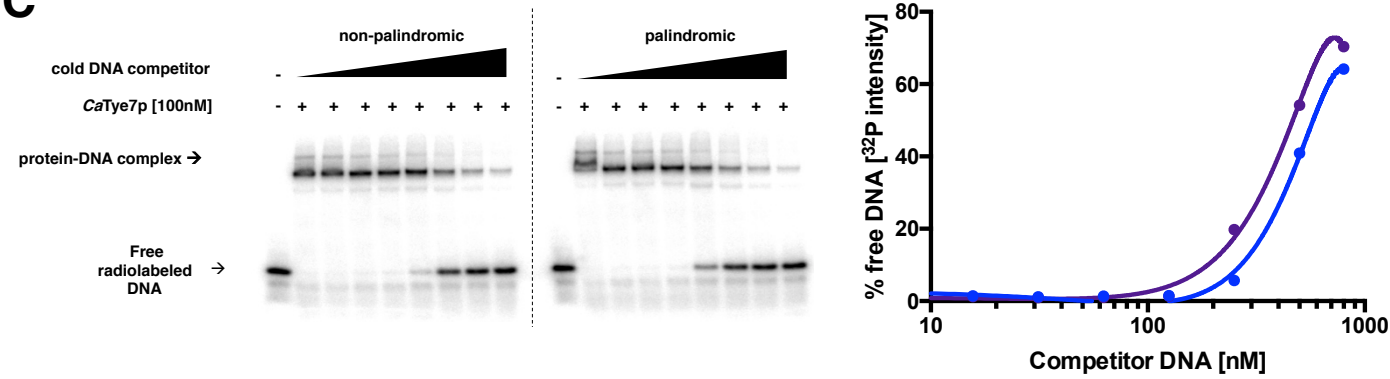

Supplement: S8 Fig — (A) Full sequences of the two DNA fragments evaluated. (B and C) Gel shift competition assays for the CaHms1 (B) and CaTye7 (C) proteins. Assays were carried out as described in the legend to Fig 4B. Quantification of competition assays is shown to the right; best-fit curves are included. Notice that the two proteins show opposing DNA binding preferences. (PDF) [file pgen.1007884.s015.pdf]
