# Supplementary material for: Diversification of DNA binding specificities enabled SREBP transcription regulators to expand the repertoire of cellular functions that they govern in fungi
Source: PLoS Genet. 2018 Dec 31;14(12):e1007884. doi: 10.1371/journal.pgen.1007884 (PMC6329520; doi:10.1371/journal.pgen.1007884)

S3 Fig.

A

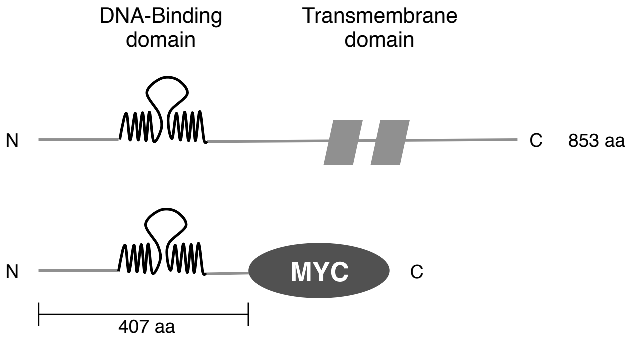

B

ORF	Gene name	Peak value - $\log_{10}$ p-value	Fold enrichment	Motif score
orf19.921	*HMS1*	61,27	5,17	4,1
orf19.3794	*CSR1*	42,25	4,49	3,9
orf19.3549	*CDC21*	34,07	3,45	3,2
orf19.3337		26,05	2,94	3,4
orf19.2333		50,89	4,16	4,8
orf19.4941	*TYE7*	20,14	2,97	3,1
orf19.6736		24,50	2,48	2,8
orf19.4167		35,59	3,88	3,3
orf19.2723	*HIT1*	32,56	3,24	3,2
orf19.4309	*GRP2*	25,16	3,02	3,8
orf19.7502		25,60	2,82	4,6
orf19.3261		17,48	2,29	4,2
orf19.7561	*DEF1*	32,49	3,29	-
orf19.610	*EFG1*	25,80	3,02	3,1

C

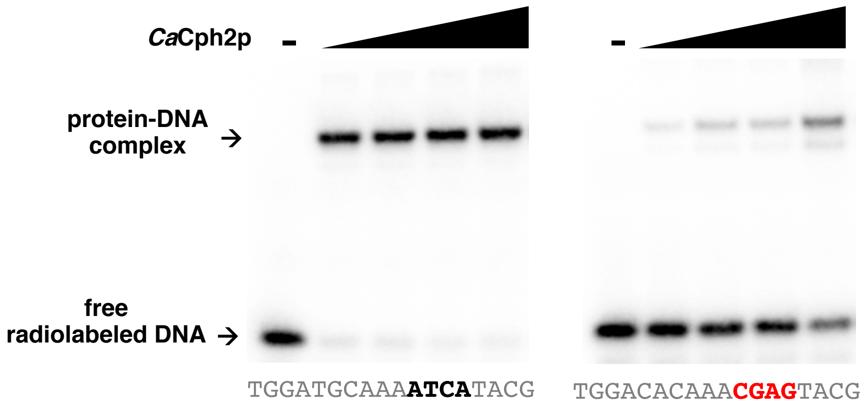

Supplement: S3 Fig — (A) Schematic representation of the CaCph2-MYC construct used for ChIP. (B) List of DNA regions occupied by CaCph2p based on our ChIP-Seq experiment. The DNA motif derived from the ChIP data was used to calculate motif scores at peak locations. (C) Gel shit assay probing the binding of the purified CaCph2 protein (0, 0.0012, 0.006, 0.02 and 0.1 nM) to the indicated P32-labeled DNA fragment (taken from the upstream intergenic region of ORF19.921) which harbors an instance of the putative Cph2 motif (in black). DNA binding is strongly reduced when point mutations (in red) are introduced in the binding site. (PDF) [file pgen.1007884.s010.pdf]
