# Supplementary material for: Diversification of DNA binding specificities enabled SREBP transcription regulators to expand the repertoire of cellular functions that they govern in fungi
Source: PLoS Genet. 2018 Dec 31;14(12):e1007884. doi: 10.1371/journal.pgen.1007884 (PMC6329520; doi:10.1371/journal.pgen.1007884)

S6 Fig.

A

Anc4 Alignment: msaprobs Outsider: Pho4

K (1.0) K (1.0) T (1.0) A (1.0) H (1.0) N (1.0) M (1.0) I (1.0) E (1.0) K (1.0) R (1.0) Y (1.0) R (1.0) T (1.0) N (1.0) L (1.0) N (1.0) D (1.0) K (1.0) I (1.0) A (1.0) A (1.0) L (1.0) R (1.0) D (1.0) S (1.0) V (1.0) P (1.0) S (1.0) A (1.0)
H (1.0) K (1.0) L (1.0) N (1.0) K (1.0) A (1.0) T (1.0) V (1.0) L (1.0) S (1.0) K (1.0) A (1.0) T (1.0) E (1.0) Y (1.0) I (1.0) H (1.0) H (1.0) L (1.0) E (1.0) K (1.0) R (1.0) V (1.0) K (1.0) R (1.0)

Anc5 Alignment: msaprobs Outsider: Pho4

K (0.997) K (0.996) T (0.962) A (0.998) H (1.0) N (1.0) M (1.0) I (1.0) E (1.0) K (1.0) R (1.0) Y (1.0) R (1.0) T (1.0) N (1.0) L (0.999) N (1.0) D (1.0) K (1.0) I (1.0) A (0.895) A (0.984) L (1.0) R (1.0) D (1.0) A (1.0) V (1.0) P (1.0) S (0.986) A (1.0)
R (0.952) K (1.0) L (1.0) N (1.0) K (1.0) A (0.996) T (1.0) I (1.0) L (1.0) S (0.999) K (1.0) A (1.0) T (1.0) E (1.0) Y (1.0) I (1.0) R (0.664) H (1.0) L (1.0) E (1.0) R (0.458) K (0.945) N (1.0) K (0.892) K (0.401)

Anc5 Alignment: muscle Outsider: Pho4

Q (0.999) K (0.994) L (0.607) A (0.99) H (1.0) N (1.0) K (0.892) I (0.999) E (1.0) K (0.998) K (0.999) Y (1.0) R (1.0) I (0.957) N (1.0) I (1.0) N (1.0) T (0.974) K (0.999) I (0.989) A (0.606) K (0.519) L (1.0) Q (0.995) K (0.662) L (0.574) I (0.975) P (1.0) W (1.0) S (0.88)
T (0.755) K (0.999) L (0.954) N (0.989) K (1.0) S (1.0) I (0.615) I (0.999) L (1.0) E (0.532) K (0.995) A (1.0) V (0.568) E (0.729) Y (1.0) I (1.0) L (0.563) Y (0.939) L (1.0) Q (0.999) N (0.56) N (0.973) E (0.968) R (0.361) L (0.467)

Anc5 Alignment: muscle Outsider: Pho4 (Mouse and Human SREBP1 and SREBP2 included)

K (0.96) K (0.998) T (0.987) A (0.994) H (1.0) N (1.0) A (0.965) I (0.995) E (1.0) K (0.802) R (1.0) Y (1.0) R (1.0) N (0.998) N (1.0) I (1.0) N (1.0) D (0.997) R (0.884) I (1.0) A (0.897) A (0.992) L (1.0) R (0.975) D (0.839) A (0.824) V (1.0) P (1.0) A (0.917) A (0.999)
T (0.952) K (1.0) L (0.999) N (0.993) K (1.0) A (0.999) T (0.994) I (0.934) L (1.0) S (0.79) K (1.0) A (0.998) T (0.996) E (0.998) Y (1.0) I (1.0) Q (0.522) H (0.792) L (1.0) K (0.851) K (0.523) R (0.986) N (0.699) K (0.711) R (0.684) N (0.952) A (0.26) R (0.555)

B

```
Anc 5.1  QKTAHNAIEKRYRTNINDKIAALRDAVPWLRIAAGCDDVSIADLEGLTPATKLNKATILSKATEYIKYLQKRNKR
Anc 5.2  Q.....A..RR..T.............................................................
Anc 5.3  K.....M..KR..N.............................................................
Anc 5.4  K.....M..RR..N.............................................................
Anc 5.5  Q.....A..KK..T.............................................................
Anc 5.6  Q.....A..RK..T.............................................................
Anc 5.7  K.....M..KK..N.............................................................
Anc 5.8  K.....M..RK..N.............................................................
```

C

**palindromic E-box**	TCTGGCCAACA**TCA**GG**TGA**ACGCAGGAAAA
**mutant DNA**	TCTGGCCAACAT**TCTTT**AAACGCAGGAAAA

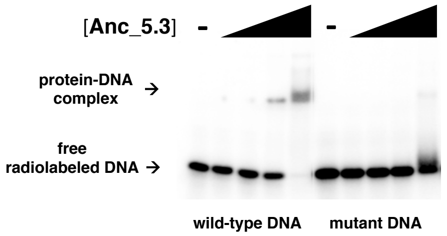

**non-palindromic site**	ACAAAAAAAAAA**TCA**GG**CCA**TTTGTAATACT
**mutant DNA**	ACAAAAAAAAAAT**TCTAAA**ATTTGTAATACT

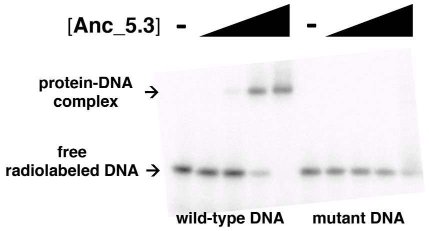

Supplement: S6 Fig — (A) Phylobot output displaying the probabilities of the given residues at each position of the reconstructed ancestors. Three reconstructions applying different alignment methods were used to infer the sequence of the “oldest” ancestor (Anc5). (B) Amino acid sequences of eight different versions of Anc5 that were synthesized and tested in in vitro gel shift experiments. Anc5.3 showed the highest affinity for DNA; hence it was selected for further characterization. Dots in the alignment represent the same amino acid residue written at the top of the column. (C) Gel shift assays testing the binding specificity of the purified Anc5 protein (0, 6.25, 25, 100 and 400 nM) towards the palindromic (left) and the non-palindromic (right) DNA sequences. Point mutations (in red) introduced in the DNA fragment abolished binding. (PDF) [file pgen.1007884.s013.pdf]
